# Supplementary material for: Workplace gender-based violence among female staff in public higher education institutions in eastern Ethiopia: Institution-based cross-sectional study
Source: PLOS Glob Public Health. 2024 Jul 26;4(7):e0003489. doi: 10.1371/journal.pgph.0003489 (PMC11280138; doi:10.1371/journal.pgph.0003489)
Supplement: Attachment — Submitted filename: marked up GBV study.pdf [file pgph.0003489.s004.pdf]

PLOS Global Public Health

Workplace gender-based violence among female staff in public higher education institutions, eastern Ethiopia: Institution-based cross-sectional study

--Manuscript Draft--

**Manuscript Number:**	PGPH-D-23-01225
**Article Type:**	Research Article
**Full Title:**	Workplace gender-based violence among female staff in public higher education institutions, eastern Ethiopia: Institution-based cross-sectional study
**Short Title:**	Workplace gender-based violence in public higher education institutions of Ethiopia
**Corresponding Author:**	Agumasie Semahegn<br>Haramaya University School of Nursing and Midwifery<br>Harar, ETHIOPIA
**Order of Authors:**	Agumasie Semahegn
	Mowlid Abdi Ali
	Wondimye Ashenafi
	Henok Legesse
**Keywords:**	Gender-based violence; female staff; Higher Educational Institutions; Ethiopia
**Abstract:**	Introduction: Gender-based violence in workplaces is a major public health challenges and human rights violation worldwide. Yet there is a paucity of evidence on gender-based violence in higher educational institutions (HEIs) or so called Universities. The main aim of this study was to asses workplace gender-based violence and its associated factors among female staff in public HEIs in eastern Ethiopia.<br><br>Methods: Institutional-based cross-sectional study design was conducted among female staff working in public HEIs in Eastern Ethiopia. A total of 391 female staff were recruited using systematic sampling technique from three HEIs. Data were collected using adapted and pre-tested self-administered questionnaire that were facilitated by trained female data collectors. Collected data were entered into EpiData and exported to SPSS for cleaning and analysis. Descriptive statistics, binary and multivariable logistic regression analysis were carried out to compute frequencies and adjusted odds ratio (AOR) with 95% confidence interval (CI).<br><br>Results: Prevalence of workplace gender-based violence among female staff was 63.1% (95% CI: 58 – 68%). Being in the age group (18–34 years) (AOR:2.3, 95% CI: 1.11-4.91), being single (AOR:3.2, 95% CI: 1.07-9.74), divorced (AOR:3.7, 95% CI: 1.08-12.39), attained Bachelor Degree (AOR: 0.46, 95%: 0.24-0.90), work in night shift (AOR: 5.5, 95%CI: 1.65-15.77), not aware of the reporting procedures (AOR: 2, 95%CI: 1.23-3.33), and worried of violence (AOR: 1.71, 95%CI: 1.02-2.86), were the factors significantly associated with workplace gender-based violence against female staff in the public HEIs.<br><br>Conclusion: The prevalence of workplace gender-based violence among female staff working in the selected public HEIs was found to be unacceptably high. We suggest awareness- raising campaigns against gender-based violence, enforcing existing policies and procedures, orentation to employees, safe guarding night shifts workers, and support service for victims should be implemented by key stakeholders.
**Additional Information:**	
**Question**	**Response**
**Financial Disclosure**	Haramaya University for financial support

Powered by Editorial Manager® and ProduXion Manager® from Aries Systems Corporation

Enter a financial disclosure statement that describes the sources of funding for the work included in this submission and the role the funder(s) played. This includes grants and any commercial funding of the work or authors.<br><br>This statement will be typeset if the manuscript is accepted for publication.<br><br>*Please review the submission guidelines and the instructions link below for detailed requirements and guidance.*	
**Competing Interests**<br><br>On behalf of all authors, disclose any competing interests that could be perceived to bias this work.<br><br>This statement will be typeset if the manuscript is accepted for publication.<br><br>*Please review the instructions link below and PLOS Global Public Health's competing interests policy to determine what information must be disclosed at submission.*	All authors declare that have no competing interest
**Data Availability**<br><br>Before publication, Authors are required to make fully available and without restriction all data underlying their findings. Please see our PLOS Data Policy page for detailed information on this policy.<br><br>A **Data Availability Statement**, detailing where the data can be accessed, is required at first submission. Insert your Data Availability Statement in the box below.	All related data are presented fully within the manuscript, and available up on reasonable request to the lead author (MAA: mowlid257@gmail.com) and the corresponding author (AS: agucell@yahoo.com)

Please see the data reporting section of our submission guidelines for instructions on what you need to include in your Data Availability Statement.<br><br>This statement will be typeset if the manuscript is accepted for publication.<br><br>*PLOS allows rare exemptions to address legal and ethical concerns. If you have legal or ethical restrictions, please use the box below to detail these in full sentences for the Journal team to consider.*	

Manuscript Click here to access/download;Manuscript;Manuscript.docx

Workplace gender-based violence among female staff in public higher education institutions, eastern Ethiopia: Institution-based cross-sectional study

Mowlid Abdi Ali[1], Agumasie Semahegn [2,3*], Wondimye Ashenafi[1,4], Henok Legesse [2]

Affiliation

[4]School of Public Health College of Health and Medical Sciences, Haramaya University, Harar, Ethiopia

[2]School of Nursing and Midwifery, College of Health and Medical Sciences, Haramaya University, Harar, Ethiopia

[3]Department of Population, Family and Reproductive Health, School of Public Health, University of Ghana, Accra, Ghana

[4] National Data Management Center for Health (NDMC), Ethiopian Public Health Institute (EPHI), Addis Ababa, Ethiopia

Email

MAA: mowlid257@gmail.com

AS: agumas04@gmail.com

WA: wondtg@gmail.com

HL: henok_legesse@yahoo.com

Correspondence *: Agumasie Semahegn (agumas04@gmail.com); School of Nursing and Midwifery, College of Health and Medical Sciences, Haramaya University, Harar, Ethiopia, and Department of Population, Family and Reproductive Health, School of Public Health, University of Ghana, Accra, Ghana

Abstract

Introduction: Gender-based violence in workplaces is a major public health challenges and human rights violation worldwide. Yet there is a paucity of evidence on gender-based violence in higher educational institutions (HEIs) or so called Universties. The main aim of this study was to asses workplace gender-based violence and its associated factors among female staff in public HEIs in eastern Ethiopia.

Methods: Institutional-based cross-sectional study design was conducted among female staff working in public HEIs in Eastern Ethiopia. A total of 391 female staff were recruited using systematic sampling technique from three HEIs. Data were collected using adapted and pre-tested self-administered questionnaire that were facilitated by trained female data collectors. Collected data were entered into EpiData and exported to SPSS for cleaning and analysis. Descriptive statistics, binary and multivariable logistic regression analysis were carried out to compute frequencies and adjusted odds ratio (AOR) with 95% confidence interval (CI).

Results: Prevalence of workplace gender-based violence among female staff was 63.1% (95% CI: 58 – 68%). Being in the age group (18–34 years) (AOR:2.3, 95% CI: 1.11-4.91), being single (AOR:3.2, 95% CI: 1.07-9.74), divorced (AOR:3.7, 95% CI: 1.08-12.39), attained Bachelor Degree (AOR: 0.46, 95%: 0.24-0.90), work in night shift (AOR: 5.5, 95%CI: 1.65-15.77), not aware of the reporting procedures (AOR: 2, 95%CI: 1.23-3.33), and worried of violence (AOR: 1.71, 95%CI: 1.02-2.86), were the factors significantly associated with workplace gender-based violence against female staff in the public HEIs.

Conclusion: The prevalence of workplace gender-based violence among female staff working in the selected public HEIs was found to be unacceptably high. We suggest awareness- raising campaigns against gender-based violence, enforcing existing policies and procedures, orentation to employees, safe guarding night shifts workers, and support service for victims should be implemnted by key stakeholders.

Keywords: Gender-based violence, female staff, Higher Educational Institutions, Ethiopia

Introduction

According to the 1993 United Nations (UN), gender-based violence is defined as "any act of violence that results in, or is likely to result in, physical, sexual, or mental harm or suffering to women, including threats of such acts, coercion or arbitrary deprivation of liberty, whether occurring in public or in private life" (1). Violence against women at workplace perhaps is the most pervasive, but less recognized human rights abuse in the world (1, 2). Workplace gender-based violence (GBV), in its different forms (physical, sexual and psychological) is worldwide problem that affect large number of women at their work sites (2, 3)(4, 5). Majority of women reported for being not safe and victim of different forms of workplace GBV (5, 6).

Workplace GBV is recognized as a severe human rights abuse and major public health challenge with substantial consequences on physical, mental, and reproductive well beings of female staff (7-12). Globally, 38% of women were murdered by their intimates (1), and the extent of workplace GBV ranging from 10% to 69% worldwide (4), and huge inequities found across regions. The prevalence of GBV was 20% in the Western Pacific, 22% in Europe, 33% in the WHO African region (1), and 67.7% in sub-Saharan Africa that include Ethiopia (9). Workplace GBV in higher education setting is alarmingly rising and almost becoming a growing concern (13). As a result of the unsafe and male dominancy in the HEIs, female staff are at increased risk to workplace GBV (14). However, the causes of workplace GBV are multi-dimensional where the interplay of social, economic, cultural, political and religious factors affects the victimization and perpetration. Besides, senior male co-workers are the main perpetrators at workplaces due to power imbalance (15-17).

Workplace GBV is a significant public health issue and a human rights agenda in Ethiopia. A systematic review and meta-analysis in Ethiopia revealed that the workplace violence is high and it is exceptionally high among female university (HEIs) staff (18). However, there is a paucity of evidence on factors influencing workplace GBV which could be the main reason for the poor implementation of workplace violence prevention and control programs in Ethiopia (18). Therefore, we conducted an institution-based study that aimed at assessing workplace GBV and its associated factors among female staff in public HEIs in eastern Ethiopia.

Materials and methods

Study setting

The study was conducted in three public universities or HEIs in the eastern part of Ethiopia, namely: Haramaya University, Dire Dawa University and Jigjiga University, from December 1 to 30 December, 2022. Haramaya University is located in Haramaya town, eastern Ethiopia that is the second oldest Public HEIs in Ethiopia. It was established in 1954 as 'Alemaya College of Agriculture' under Addis Ababa University until 1985. Currently, Haramaya University has widely been engaged in the expansion and diversification of undergraduate, graduate and postgraduate trainings (19). Dire Dawa University (DDU) is a public university located in Dire Dawa, Ethiopia that is founded in 2007.as second-generation university, and has six colleges (20). Similarly, Jigjiga University (JJU) is one of the public University that was established in 2007 second-generation universities in Ethiopia. It's found in Jigjiga, the capital of the Somali Region, Ethiopia. Jigjiga is located, 635 kilometers from Addis Ababa, eastern Ethiopia. It has 8 faculties; it also offers both post and under graduate program (21). Most of the populations are pastoralists as well as engaged in trade, cultivation of cash crops, and in transitive life (22).

Study design and participants

An institutional-based cross-sectional study was conducted on the selected public universities. All female staff whose age above 18 years and who had been working for at least 6 months in the selected universities were included in the study. Nevertheless, female staff who were on annual leave, maternity leave and severe health conditions during data collection period were excluded from the study.

Sampling procedures

Sample size was determined by using single population proportion formula considering parameters of workplace violence prevalence (63.8%) in Nigeria (17), 95% significance level, 1% margin of error, and adding 10% potential non-response compensation. Finally, the sample size was 391. Then the calculated sample size was proportionally allocated to the number of female staff at selected public HEIs (**Fig 1**). The sampling frame was constructed by listing all female staff working in the selected public HEIs. Actual study participants were selected using systematic sampling. The first study participant from each university was

selected by using lotters method and then every participant (kth) was selected according to the respective sampling interval until the sample size was fulfilled for each university.

Fig 1: Schematic presentation of sampling procedures

Data collection procedure

Data were collected using self-administered structured questionnaire that was adapted from International Labor Office (2), country based survey questionnaire on workplace violence and from “UNICEF: Ending gender based violence survey questionnaire” on HEIs (23). The questionnaire contains: socio-demographic and workplace characteristics (12 items), Institutional/contextual characteristics (9 items), GBV assessing questions for psychological violence (8 items), physical violence (4 items), and sexual violence and/or harassment (14 items) and responses toward the incidents (5 items). It was first written in English and then translated to Amharic, Afaan-Oromo and Somali language and was re-translated to English by language experts to ensure consistency. Data collection were facilitated by 10 trained female Nurses. Four data collectors for Haramaya University, three for Jigjiga University, three for Dire Dawa University were assigned to facilitate the self-administered questionnaire distribution, clarification as deemed necessary and re-collection. Training was provided to data collectors on the study objectives, sampling, consent, data privacy, checking for data clarity and completeness by the principal investigator. Then data collectors taken informed consent from the study participants.

Outcome variable measurements

Workplace GBV against female staff at HEIs was measured as a composite variable using the three very common types of workplaces GBV, namely physical violence, psychological violence and sexual violence and/or harassment. So, in this study, workplace GBV was considered if a female staff (15-49 years) reported experienced of at least one forms of violence (physical, psychological, or sexual violence) by male co-worker, supervisor/boss, visitor or strange person at their workplace in the past 12 months (10). Psychological violence includes either one of: intentional use of power, including threat of physical force, act of verbal abuse including insults and bullying or mobbing. Physical violence was assessed when female staff answered “yes” to one of the items: slapping, kicking, beating with any object, cutting, pushing, shoving, throwing, strangling, grabbing or pulling, and shooting against female staff. Sexual violence is defined as acts of violence at workplace that are done on a female staff by intentional use of physical force or power, intimidation or

threatening (making to fear) to have sex or to engage in acts of sex without the consent. It includes completed rape, attempted rape and sexual harassment.

Data processing and analysis

Collected data were entered into Epi-Data 3.1 and exported to IBM SPSS version 25 for cleaning and analysis. Descriptive statistics was carried to determine the proportion, mean with standard deviations of explanatory variables and prevalence of workplace violence. Binary and multiple logistic regression analyses were performed to examine the association between the independent variables and workplace GBV. Explanatory variables with a p-value < 0.25 in binary logistic regression analysis were included in the multivariable logistic regression analysis. The Hosmer-Lemeshow test was used for goodness of fit for the final model fitness. The adjusted odds ratio (AOR) with 95% confidence interval (CI) at P-Value of < 0.05 was considered to declare significance association between explanatory variables and outcome variable.

Results

Socio-demographic characteristic of study participant

A total of 385 study participants completed the questionnaire (Fig.1) which made the response rate 98.5% (385/391). The mean age for participants was 32.39 (SD±8.65) years. Nearly half of them were married (45.2%, n=174) and belonging to Orthodox Christianity (48.3%, n=186). Forty percent (n=151) of the study participants were bachelor degree holders (Table 1).

Table 1. Socio-demographic characteristics of the study participants in HEIs, eastern Ethiopia, 2022 (n=385)

**Variables**	**Categories**	n	**%**
Age	18-34	246	63.9
	≥35	139	36.1
Marital status	Single	146	37.9
	Married	174	45.2
	Divorced	43	11.2
	Widowed	22	5.2
Religion	Muslim	107	27.8
	Orthodox	186	48.3
	Protestant	78	20.3
	Others*	14	3.6
Ethnicity	Oromo	138	35.8

	Amhara	160	41.6
	Somali	61	15.8
	Others**	26	6.8
Educational status	Secondary school	35	9.1
	Diploma level	113	29.4
	Bachelor’s degree	154	40
	Master’s degree and above	83	21.5
Salary level (Ethiopian Birr)	1100-2344	117	30.4
	2799-5358	117	46.0
	6193-10150	71	18.4
	≥10150	20	5.2
Service years	1-4	163	42.3
	5-9	146	37.3
	≥10	76	19.7
Residence	Inside campus	70	18.2
	Outside campus	315	81.8

**Others include: Catholic, Waqefata, Jehovah’s Witness, un-affiliated. **Others include: Tigray, Gurage, Sidama, Harari*

Workplace related characteristics

Out of 385, nearly half (47.5%, n=183) of them were from Haramaya University. Majority (90.4%) of them did not work in night shifts, and 71.7% of them did not know how to report violence. Slightly more than three-fourth (76.3%, n=293) of them were administrative staff, and nearly all of them (96.4%) had permanent employment (Table 2).

Table 2: Institutional related characteristics of female staff working in public HEIs of eastern Ethiopia, 2022 (n=385)

**Variables**	**Categories**	n	**%**
Institution	HU	183	47.5
	JJU	98	25.5
	DDU	104	27
Staff category	Academic	92	23.9
	Non- Academic	293	76.3
Number of co-workers	1-4	307	79.7
	5-9	45	11.7
	≥10	33	*8 .6*
Night shift	Yes	37	9.6
	No	348	90.4
Employment status	Temporary	14	3.6
	Permanent	371	96.4
Reporting procedures	Yes	109	28.3
	No	276	71.7
Institutional policy	Yes	235	61
	No	150	39
Worried of violence	Not worried	134	34.8
	Little worried	50	13

	Moderately worried	143	37.1
	Much worried	58	15.1

Workplace gender-based violence against female staff

Female staff experience of workplace GBV was computed as a composite measure. By considering any forms of psychological, sexual and physical violences, the prevalence of workplace GBV against female staff in HEIs was 63.1% (95% CI: 58-68%) (Fig 2).

Fig **2**: Workplace GBV among female staff in public HEIs, eastern Ethiopia, 2022 (n=385)

Perpetrators of the workplace GBV

One-third (34.8%) of female staff perpetrated by their supervisor/boss, followed by 20.8% of them perpetrated by co-workers (Fig. 3).

Fig **3**: Perpetrators of workplace GBV among female staff in public universities of eastern Ethiopia, 2022 (n=385)

More than a quarter (28.1%) of female staff who had experienced workplace GBV reported that reduced productivity due to GBV at workplace. More than one-in-ten (11.7%) of them stay off work after the incident. One-in-five (20.5%) of them claimed that they were emotionally exhausted after experienced violence at their workplace.

Factors associated with workplace gender-based against female staff

In the binary logistic regressions, a total of 20 independent variables were included. Then only 11 variables namely; marital status, educational status, residence, work experiences, number of co-workers, age, staff category, night shift, reporting procedure, worried of violence and institutional policy were found to be significant with a p-value of < 0.25 in the bivariate analysis (Table 3). In the final multivariable logistic regression model, six variables remained significantly associated with workplace GBV. Being single (AOR: 3.2, 95% CI: 1.07-9.74) and separated/divorced (AOR: 3.7, 95% CI: 1.08-12.39) were associated with increased odds of WPGBV as compared to being currently married. Moreover, being in age group of 18-34 years (AOR: 2.3, 95% CI: 1.11-4.91), working in the night shift (AOR:5.5, 95% CI: 1.65-15.77), being not aware of the existence of reporting procedures (AOR: 2.0, 95% CI: 1.23-3.33) and being worried about violence (AOR: 1.71, 95% CI: 1.02-2.86) were also associated with increased odds of WPGBV as compared with their counterparts. On the other hand, the odds of experiencing workplace GBV were decreased by 54% among female

staff with BSc degrees compared to female staff with Masters and above degree (AOR:0.46, 95% CI:0.24-0.90) (Table 3).

Table 3. Factors associated with GBV among female staff working in public HEIs in the eastern Ethiopia, 2022 (n=385)

**Variable**	**Category**	**Workplace GBV**		**COR (95%CI)**	**AOR (95% CI)**
		**Yes**	**No**		
Marital status	Single	110	36	4.4(1.74-11.2)	3.2(1.07 -9.74) *
	Widowed	9	13	2.1(0.67-4.12)	1.4(0.49-4.17)
	Divorced	31	12	3.7(1.31-10.1)	3.7(1.08-12.39) *
	Married	93	81	1	1
Educational Status	≤Secondary	23	12	0.61(0.26-1.44)	0.74(0.28- 1.97)
	Diploma	76	37	0.65(0.34-1.24)	0.86(0.42-1.78)
	BSc	81	73	0.35(0.19-0.64)	0.46(0.24-0.90) *
	≥MSc	63	20	1	1
Residence in campus	Inside	49	21	1.45(0.83-2.55)	1.5 (0.79-2.87)
	Outside	194	121	1	1
Work experience	1-4	95	68	1.02(0.59-1.76)	0.73(0.39-1.37)
	5-9	104	42	1.8(1.01-3.22)	1.32(0.70-2.52)
	≥10	44	32	1	1
Co-workers'	1-4	183	124	0.4(0.17-0.94)	0.61(0.23-1.59)
	5-9	34	11	0.8(0.28-2.44)	1.20(0.38-3.99)
	≥10	26	7	1	1
Age	18-34	171	75	2.7 (1.4-5.1)	2.30(1.11-4.91) *
	≥35	50 22	66	1	1
Staff category	Academic	63	29	1.4 (0.8-2.3)	1.30(0.74-2.43)
	Administrative	180	113	1	1
Night shift	Yes	33	4	0.2 (0.1-0.5)	5.5(1.65-15.77) *
	No	210	138	1	1
Reporting procedure	Yes	188	88	1	1
	No	55	54	2.1 (1.33-3.29)	2(1.23-3.33) *
Worried of violence	Worried	94	40	0.69(0.1-0.44)	1.71(1.02-2.86) *
	Not worried	149	102	1	1
Institutional policy	Yes	101	49	1	1
	No	142	93	0.7(0.48-1.14)	0.79 (0.49-1.29)

**Significantly associated at a p-value of <0.05*

Discussion

This study determined the prevalence of workplace GBV and associated factors among female staff in public higher education institutions. Nearly two-third (63.1%, 95%CI: 58–68%) of female staff at HEIs experienced workplace GBVs during the last 12 months. This finding is in line with the study conducted in Nigeria, where prevalence of workplace GBV among female staff was (63.8%) (24), and the study conducted in Iran (63.2%) (25). However, it is higher than the findings in Nepal (49.5%) (6), in Turkey (44.7%) (8), in

Rwanda (58.5%) (10), in Ethiopia (35.1%) (26). This discrepancy is might be due to nature of the study, cultural, socio-economic difference and it might also be due to violence prevention regulations, strategies and policies existed in those countries. The findings of this study is lower than the finding of study conducted in China (77.5%) (27), and a comparative study on gender based violence in educational institutions of Kenya (70%) (28).

In the present study psychological violence is the most common forms of workplace GBV (57.7%). This findings is similar with the study conducted in Nigeria (29), Gambia (30). Different forms of psychological violence were faced by the female staff once and more than twice times in last twelve months in higher education institutions. In the present study sexual violence found to be (14.5%), this finding is similar with study conducted in Nepal (6). Sexual violence of this findings is much lower than the study conducted in Ethiopia that have reported 49.1% in Bahir Dar City 49.1% (31), 39.2% in Dessie City (32), 56.4% in Harar (33). The finding of this study showed that sexual violence high in younger age female staff compared to older age, the study is similar with study conducted in Ethiopia (34). Similarly, the findings of the present study revealed that (28.3%) of female staff were experienced physically violence which is similar with study conducted in Zimbabwe (35), study conducted in Harar town, Ethiopia, where nearly one-third of women experienced at least one form of physical violence in Ethiopia (33)(36) and in United Kingdom (14).

According to the findings of the current survey having bachelor degree significantly associated with less experience of workplace GBV. This result is in line with study conducted Taiwan (37) and in Nigeria among female staff university also support this finding (24). Different findings might indicate that education protects women against disrespectful or inappropriate behavior at work, including in academics and the findings (38, 39). The findings of this study revealed that female staff between (18-34) years experienced more GBV at workplace. This result is similar with the study conducted in Ethiopia (3). Furthermore, our findings showed that as age increased the risks of GBV at workplaces among university female staff decreases. This findings may be that people's reverence for old age in various Ethiopia cultures discourages the exhibition of violence against older female staff, this finding is supported with study conducted in Nigeria (24), as well as study conducted in China (40).

In the present study, participants work Night shifts was 5.5 times were more likely to experiences workplace GBV. This findings was similar to the study conducted in Egypt (41) and the study conducted in Ethiopia among female staff at Mekelle University (42), public hospitals of Eastern Ethiopia (43). The odds of workplaces GBV were higher in those females who were not-aware of reporting procedure compared to those female staff who aware reporting procedures. These factors were noted in a study conducted in Saudi Arabia (44) and study conducted in Ethiopia (36). The finding of this study demonstrated that being worried about violence at workplace were substantially associated with Workplaces GBV. These factors were in line with the study conducted in Brazil (45). This might be indicating that those who were worried of violence were working in a predisposed work environment.

Strength and weakness of the study

The study used a validated and comprehensive tool that encompasses the magnitude, nature and effect of workplace GBV in a relatively broader fashion. But there is the possibility of response biases since research evidence suggests that female staff tend not to report workplace of GBV experience for fear of stigma or retribution. It may also be prone to recall-bias. The causal relationship between explanatory variables and workplace GBV may be limited due to temporality challenges in the cross-sectional study design.

Conclusions

The prevalence of workplace GBV among female staff at higher education institution, in eastern Ethiopia was found to be high. Female staff experience of forms of GBV in the workplace was significantly associated with their age, lower academic qualification, marital status, reporting procedures and work experience of 4-9 years. It was mainly perpetrated by supervisors. Therefore, it's recommended to establish workplace GBV committee that would track the incidents, provide guidelines and make sure all corrective measures were taken and communicated properly.

Acronyms and abbreviations

AOR: Adjusted Odds Ratio; CI: Confidence Interval; COR: Crude Odds Ratio; GBV: Gender-Based Violence; HEI: Higher Education Institutions; KII: Key Informant Interview; UN: United Nations; VAW: Violence Against Women; WHO: World Health Organization; WPV: Workplace Violence; WPGBV: Workplace Gender-Based Violence

Declaration

Ethical consideration

The study protocol was reviewed and approved by Institutional Health Research Ethical Review Committee College of Health and Medical Science, Haramaya University, (Refn.: IHRERC/176/2022). A formal letter was submitted for each university administrations and permission was granted to conduct the data collection. The study was conducted in accordance with the declaration of Helsinki's. Informed verbal and written consent were obtained from each study participant in voluntary basis to be included in the study. Informed consent from participant/legal guardian and assent from them were obtained from study participants whose age younger than 18 years old, and also not attended formal education. Collected data had kept confidential anonymously through de-identification of names and other personal identifiers from record/sheet., parents/guardians in case of minor study participants and legally authorized representatives in case of illiterate participants.

Competing interests: Authors have no competing interest

Consent for publication: Not applicable

Availability of data and materials: All related data are presented fully within the manuscript, and available up on reasonable request to the lead author (MAA: mowlid257@gmail.com) and the corresponding author (AS: agucell@yahoo.com).

Competing interests: All authors declare that have no conflict of interest.

Funding: Haramaya University provided the financial and administrative supports for this study.

Authors' Contributions

MAA, AS, and WA had involved since conception, study design, execution, and acquisition of data, analysis and interpretation. AS, HL and MAA drafted the manuscript. All authors revised the manuscript critically for important intellectual contents. All authors read and approved the final manuscript.

Acknowledgments

We would like to acknowledge Haramaya University for financial support, and the study participants, data collectors, supervisors, health facility administrators, and staff for their willingness to give their time and information for this study.

Authors information

(MAA & WA)[1] School of Public Health College of Health and Medical Sciences, Haramaya University, Harar, Ethiopia (AS &, HL)[2]School of Nursing and Midwifery, College, Health and Medical Sciences, Haramaya University, Harar, Ethiopia; (AS)[3]Department of Population, Family and Reproductive Health, School of Public Health, University of Ghana, Accra, Ghana;

References

1. WHO. Violence against women prevalence estimates, 2018: global, regional and national prevalence estimates for intimate partner violence against women and global and regional prevalence estimates for non-partner sexual violence against women. ISBN 978-92-4-002225-6 2021;ISBN 978-92-4-002225-6 112.
2. ILO W, WHO PJGI. Framework guidelines for addressing workplace violence in the health sector. 2002.

3. Tiruneh BT, Bifftu BB, Tumebo AA, Kelkay MM, Anlay DZ, Dachew BAJBn. Prevalence of workplace violence in Northwest Ethiopia: a multivariate analysis. 2016;15(1):1-6.
4. Garcia-Moreno C, Amin AJS, matters rh. Violence against women: where are we 25 years after ICPD and where do we need to go? 2019;27(1):346-8.
5. Adebayo O, Ugorji J, Ekeh A, Jolade E, Odoh N, Ikeme A, et al. The impact of workplace violence on nurses and nursing practice: A literature review. 2022.
6. Heise L EM, Gottemoeller M. Ending Violence against Women. Baltimore: John's Hopkins University School of Public Health; Population Information Program; 1999. Report No.: Series L, No. 11. Gender based violence at workplace: are women safe. 2014.
7. Fute M, Mengesha ZB, Wakgari N, Tessema GAJBn. High prevalence of workplace violence among nurses working at public health facilities in Southern Ethiopia. 2015;14(1):1-5.
8. Pinar T, Acikel C, Pinar G, Karabulut E, Saygun M, Bariskin E, et al. Workplace Violence in the Health Sector in Turkey: A National Study. Journal of interpersonal violence. 2017;32(15):2345-65.
9. Mingude AB, Dejene TM. Prevalence and associated factors of gender based violence among Baso high school female students, 2020. Reproductive health. 2021;18(1):247.
10. Musengamana V, Adejumo O, Banamwana G, Mukagendaneza MJ, Twahirwa TS, Munyaneza E, et al. Workplace violence experience among nurses at a selected university teaching hospital in Rwanda. 2022;41.
11. Sabbar DK, Kassim WJJPJoM, Sciences H. Relationship between Workplace Related Violence and Job Satisfaction among Nurses Staff. 2022;16(03):1018-.
12. Teshome E, Kumar S. GENDER-BASED VIOLENCE AND ITS ASSOCIATION WITH MENTAL HEALTH AMONG WOMEN WITH DISABILITIES. ISSN (Online): 0493-2137. 2022;Vol:55 Issue.
13. Mahlori XF, Byrne DC, Mabude LAJSo. Perceptions of gender-based violence among university staff: interim results. 2018;8(3):21.
14. Jones C, Skinner T, Gangoli G, Smith O, Fenton RJTSCWP. Gender-based Violence Among UK University Students and Staff: A Socio-Ecological Framework. 2020;21(21-2).
15. Esther O. GENDER-BASED VIOLENCE IN WORKPLACES: A CRITICAL EXAMINATION OF ITS IMPLICATIONS IN THE CONTEMPORARY SOCIETY. 2020;1(2).
16. Oppong D, Bannor RKJCSS. Gender and power work relationships;: A systematic review on the evidence from Africa and Asia. 2022;8(1):2031686.
17. Olaoluwa Samson Agbaje1 CKA, Joshua Emeka Umeifekwem1,, Prince Christian Iheanachor Umoke1* CCI, Tochi Emmanuel Iwuagwu1, Cylia Nkechi Iweama1, Eyuche Lawretta Ozoemena1 and Edith N. Obande‑ Ogbuinya2. Workplace gender-based violence and associated factors among university women in Enugu, South-East Nigeria: an institutional-based cross-sectional study. 2021;21(1):1-20.
18. Worke MD, Koricha ZB, Debelew GTJRh. Prevalence of sexual violence in Ethiopian workplaces: systematic review and meta-analysis. 2020;17(1):1-15.
19. Mesele TT, Dheresa M, Oljira L, Wakwoya EB, Gemeda GMJIJoWsH. Prevalence of dysmenorrhea and associated factors among Haramaya university students, eastern Ethiopia. 2022:517-27.
20. Haileyesus M, Meretu D, Abebaw EJJoE, Development S. Determinants of university staff turnover intention: The case of Dire Dawa university, Ethiopia. 2019;10(17):20-9.
21. Crump L, Mohammed Ali S, Tschopp R, Zinsstag JJOHCS. Jigjiga University One Health Initiative. 2022(2022):ohcs20220001.
22. Mureda E, Zeleke ZMJH. Characteristics and constraints of crossbred dairy cattle production in lowland areas of Eastern Ethiopia. 2008;21(47.6):33.3.
23. Ranea-Triviño B, Pajares L, Bustelo M, Pereira BCJ, PU DL. Report on Case Studies on the Effects and Consequences of Institutional Responses to Gender-based Violence along the 7Ps in Research Performing Organisations.
24. Olaoluwa Samson Agbaje1 CKA, Joshua Emeka Umeifekwem1,, Prince Christian Iheanachor Umoke1* CCI, Tochi Emmanuel Iwuagwu1,, Cylia Nkechi Iweama1 ELOaENOO. Workplace gender-based violence and associated factors among university women in Enugu, South-East Nigeria: an institutional-based cross-sectional study. 2021;21(1):1-20.
25. Nasrabadi AN, Abbasi NH, Mehrdad NJGjohs. The prevalence of violence against Iranian women and its related factors. 2015;7(3):37.
26. Birkie M, Zenebe Y, Biset G, Gebresellassie M, Mihret SJTOPHJ. Risk factors for gender-based violence among female students of Gonder Teacher'Training College, Gonder, Northwest Ethiopia: a cross-sectional study. 2020;13(1).
27. Sui G, Liu G, Jia L, Wang L, Yang G. Associations of workplace violence and psychological capital with depressive symptoms and burn-out among doctors in Liaoning, China: a cross-sectional study. BMJ open. 2019;9(5):e024186.

28. Venâncio KC, da Fonseca RM. [Women working at university restaurants: life and work conditions and gender-based violence]. Revista da Escola de Enfermagem da U S P. 2013;47(5):1016-24.
29. Iliyasu Z, Abubakar IS, Aliyu MH, Galadanci HS, Salihu HMJAjorh. Prevalence and correlates of gender-based violence among female university students in Northern Nigeria. 2011;15(3):123-33.
30. Sisawo EJ, Ouédraogo SYYA, Huang S-LJBhsr. Workplace violence against nurses in the Gambia: mixed methods design. 2017;17(1):1-11.
31. Belay HG, Liyeh TM, Tassew HA, Ayalew AB, Goshu YA, Mihretie GNJIjorm. Magnitude of gender-based violence and its associated factors among female night students in Bahir Dar City, Amhara Region, Ethiopia. 2021;2021:1-7.
32. Gebrie S, Wasihun Y, Abegaz Z, Kebede NJBwsh. Gender-based violence and associated factors among private college female students in Dessie City, Ethiopia: mixed method study. 2022;22(1):513.
33. Abubeker F, Dessie Y, Assefa N, Geleto A, Adorjan K, Abdeta TJITJoHCO, Provision,, et al. Prevalence and associated factors of gender-based violence among female students attending private colleges in Harar Town, Eastern Ethiopia. 2021;58:00469580211047197.
34. Henock A, Deyessa N, Abajobir AJJWH, Issues Care 4. Sexual violence and substance use among female students of Mizan-Tepi University, Southwest Ethiopia: A mixed method study. 2015;4(2).
35. Mukanangana F, Moyo S, Zvoushe A, Rusinga OJAjorh. Gender based violence and its effects on women's reproductive Health: the case of Hatcliffe, Harare, Zimbabwe. 2014;18(1):110-22.
36. Legesse H. Workplace Violence And Its Associated Factors Among Nurses Working In Government Hospitals Of Harari Regional State And Dire Dawa City Administration, Eastern Ethiopia: Haramaya university; 2021.
37. Yoo M, Lee S, Kang MY. Gender and educational level modify the relationship between workplace mistreatment and health problems: a comparison between South Korea and EU countries. Journal of occupational health. 2015;57(5):427-37.
38. Albezreh S, Anastario M, Ulibarrí BJ, Naimer K, Johnson K, McHale T, et al. Multiyear, Multisectoral Training Program in Kenya to Enhance Medical–Legal Processes in Response to Sexual and Gender-Based Violence. 2022;28(14):3311-30.
39. Bondestam F, Lundqvist MJEJoHE. Sexual harassment in higher education–a systematic review. 2020;10(4):397-419.
40. Jan AJIJoTiSR, Development. Violence against Women with Special Reference to Domestic Violence Act, 2005. 2019;3(3):1398-401.
41. Abou-ElWafa HS, El-Gilany A-H, Abd-El-Raouf SE, Abd-Elmouty SM, El-Sayed Hassan El-Sayed RJJoiv. Workplace violence against emergency versus non-emergency nurses in Mansoura university hospitals, Egypt. 2015;30(5):857-72.
42. Galu SB, Gebru HB, Abebe YT, Gebrekidan KG, Aregay AF, Hailu KG, et al. Factors associated with sexual violence among female administrative staff of Mekelle University, North Ethiopia. 2020;13(1):1-7.
43. Legesse H, Assefa N, Tesfaye D, Birhanu S, Tesi S, Wondimneh F, et al. Workplace violence and its associated factors among nurses working in public hospitals of eastern Ethiopia: a cross-sectional study. 2022;21(1):1-10.
44. Alkorashy HAE, Al Moalad FBJInr. Workplace violence against nursing staff in a Saudi university hospital. 2016;63(2):226-32.
45. Tsukamoto SAS, Galdino MJQ, Robazzi MLdCC, Ribeiro RP, Soares MH, Haddad MdCFL, et al. Occupational violence in the nursing team: prevalence and associated factors. 2019;32:425-32.

 Click here to access/download;Figure;Fig 1.docx

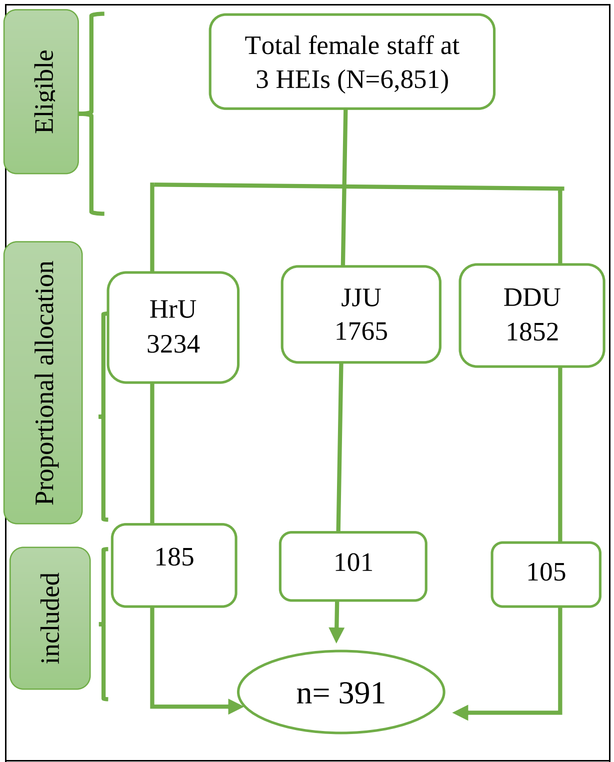

DDU: Dire Dawa University, HrU: Haramaya University, and JJU: Jigjiga University

Fig 1: Schematic presentation of sampling procedures

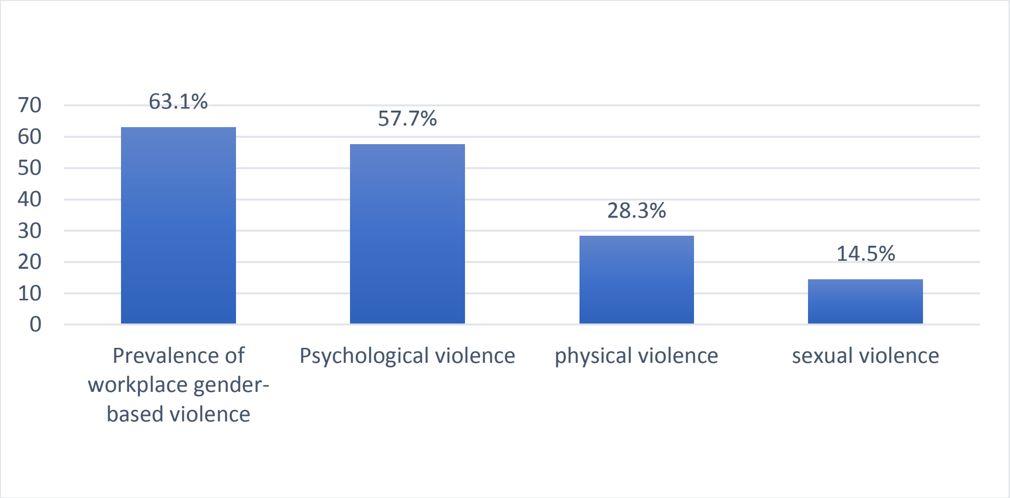

Fig **2**: Workplace gender-based violence among female staff working in public HEIs universities of eastern Ethiopia, 2022 (n=385)

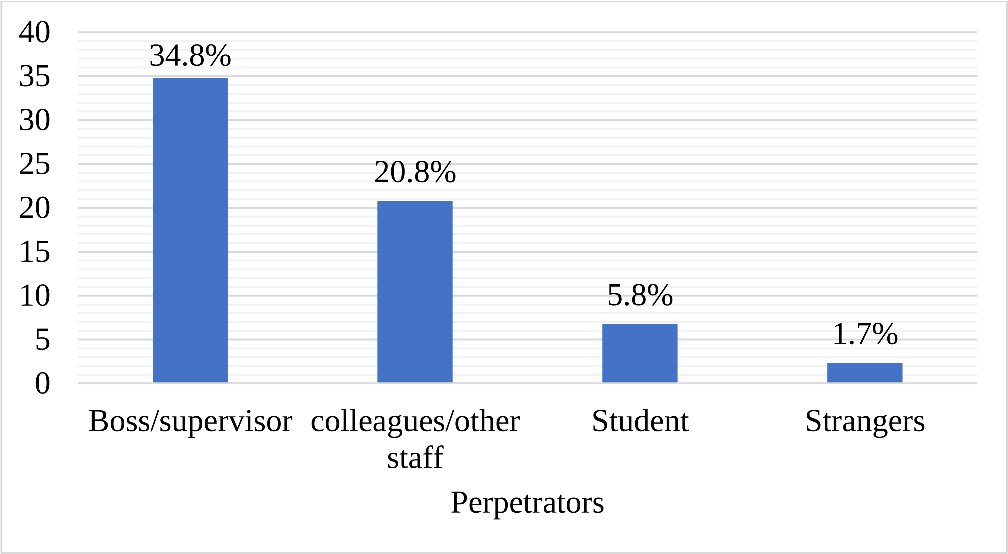

Fig **3**: Perpetrators of workplace GBV among female staff in public universities of eastern Ethiopia, 2022 (n=385)
