# Supplementary material for: Workplace gender-based violence among female staff in public higher education institutions in eastern Ethiopia: Institution-based cross-sectional study
Source: PLOS Glob Public Health. 2024 Jul 26;4(7):e0003489. doi: 10.1371/journal.pgph.0003489 (PMC11280138; doi:10.1371/journal.pgph.0003489)
Supplement: Attachment — Submitted filename: Response to Reviewers_r3.pdf [file pgph.0003489.s007.pdf]

Response to Reviewers comments

To: **PLOS Global Public Health**

Title: *Workplace gender-based violence among female staff in public higher education institutions, eastern Ethiopia: Institution-based cross-sectional study*

PLOS Global Public Health [PGPH-D-23-01225]

Dear Editor and reviewers;

We have received comments from reviewers and we fund them constructive and helpful. We have addressed the reviewers' concern point-by-point here below

Section	**Editor comment**	***Authors' response***
	In particular, I've reviewed the manuscript and found that it still suffers from many grammatical mistakes and a lack of clarity in terms of its English language. The changes made since the last version do not fix the many issues with grammar and presentation. I have therefore no choice but to send the manuscript back to you for another round of English language editing. I would suggest that you get hold of a fluent, preferably native-level English speaker to copy-edit your manuscript. If you do not know of one, you can hire professional services (see below). However you get it done, please make sure to look over the language issues properly this time.	***Many thanks for the critical review and we have dome the revision as per suggestion***
Journal Requirements:	1. Please review your reference list to ensure that it is complete and correct. If you have cited papers that have been retracted, please include the rationale for doing so in the manuscript text, or remove these references and replace them with relevant current references. Any changes to the reference list should be mentioned in the rebuttal letter that accompanies your revised manuscript. If you need to cite a retracted article, indicate the article's retracted status in the References list and also include a citation and full reference for the retraction notice	***Many thanks for the comment, we have updated the references and addressed missed components of the reference citation mainly the publisher/journal.***
	2. Please amend your detailed Financial Disclosure statement. This is published with the article. It must therefore be completed in full sentences and contain the exact wording you wish to be published.	***Thanks, we have addressed and see track change***
	a. State the initials, alongside each funding source, of each author to receive each grant.	***Thanks, we have addressed and see track change***
	b. State what role the funders took in the study. If the funders had no role in your study, please state: "The funders had no role in study design, data collection and analysis, decision to publish, or preparation of the manuscript."	**Funding is properly described**
	c. If any authors received a salary from any of your funders, please state which authors and which funders.<br><br>If you did not receive any funding for this study, please simply state: "The authors received no specific funding for this work	***Sure, we have stated our salary source and research and financial support to this research very briefly as per the standard of the journal***
	3. Please provide separate figure files in .tif or .eps format only and remove any figures embedded in your manuscript file. Please also ensure all files are under our size limit of 10MB.<br><br>For more information about figure files please see our guidelines:<br><br>https://journals.plos.org/globalpublichealth/s/figures<br>https://journals.plos.org/globalpublichealth/s/figures#loc-file-requirement	***Yes, thanks***
	4. We have noticed that you have uploaded Supporting Information files, but you have not included a list of legends. Please add a full list of legends for your Supporting Information files after the references list	***Thanks and done as pe rthe suggestion***

Many thanks!

Corresponding Author
