# Supplementary material for: Workplace gender-based violence among female staff in public higher education institutions in eastern Ethiopia: Institution-based cross-sectional study
Source: PLOS Glob Public Health. 2024 Jul 26;4(7):e0003489. doi: 10.1371/journal.pgph.0003489 (PMC11280138; doi:10.1371/journal.pgph.0003489)
Supplement: Attachment — Submitted filename: Response to Reviewers_r4.pdf [file pgph.0003489.s008.pdf]

Response to Reviewers comments

To: **PLOS Global Public Health**

Title: *Workplace gender-based violence among female staff in public higher education institutions, eastern Ethiopia: Institution-based cross-sectional study*

PLOS Global Public Health [PGPH-D-23-01225]

Dear Editor;

We have addressed the comments from s and we fund them constructive and helpful. We have addressed the reviewers' concern point-by-point here below

Section	**Editor comment**	***Authors' response***
Journal Requirements:	1. Please update your online Competing Interests statement. If you have no competing interests to declare, please state: "The authors have declared that no competing interests exist."	***Many thanks for the comment, we have updated the references and addressed missed components of the reference citation mainly the publisher/journal.***
	2. Please provide separate figure files in .tif or .eps format only and remove any figures embedded in your manuscript file. Please also ensure that all files are under our size limit of 10MB. You may leave the figure captions or legends in the manuscript.<br><br>For more information about how to convert your figure files please see our guidelines: https://journals.plos.org/globalpublichealth/s/figures	***Thanks, we have addressed all***
	3. We have noticed that you have uploaded Supporting Information files, but you have not included a list of legends. Please add a full list of legends for your Supporting Information files after the references list.	**Thank you so much all, legends are included : 4 support information files**
	4. Please review your reference list to ensure that it is complete and correct. If you have cited papers that have been retracted, please include the rationale for doing so in the manuscript text, or remove these references and replace them with relevant current references. Any changes to the reference list should be mentioned in the rebuttal letter that accompanies your revised manuscript. If you need to cite a retracted article, indicate the article's retracted status in the References list and also include a citation and full reference for the retraction notice.	***Many thanks, we have reviewed and addressed***

Many thanks!

Corresponding Author
