# Supplementary material for: Workplace gender-based violence among female staff in public higher education institutions in eastern Ethiopia: Institution-based cross-sectional study
Source: PLOS Glob Public Health. 2024 Jul 26;4(7):e0003489. doi: 10.1371/journal.pgph.0003489 (PMC11280138; doi:10.1371/journal.pgph.0003489)
Supplement: Attachment — Submitted filename: DOC-20230802-WA0001. (1).docx [file pgph.0003489.s003.docx]

PLOS Global Public Health

# Workplace gender-based violence among female staff in public higher education institutions, eastern Ethiopia: Institution-based cross-sectional study

--Manuscript Draft--

| **Manuscript Number:** | PGPH-D-23-01225 |
| --- | --- |
| **Article Type:** | Research Article |
| **Full Title:** | Workplace gender-based violence among female staff in public higher education institutions, eastern Ethiopia: Institution-based cross-sectional study |
| **Short Title:** | Workplace gender-based violence in public higher education institutions of Ethiopia |
| **Corresponding Author:** | Agumasie Semahegn  Haramaya University School of Nursing and Midwifery Harar, ETHIOPIA |
| **Order of Authors:** | Agumasie Semahegn |
|  | Mowlid Abdi Ali |
|  | Wondimye Ashenafi |
|  | Henok Legesse |
| **Keywords:** | Gender-based violence; female staff; Higher Educational Institutions; Ethiopia |
| **Abstract:** | Introduction: Gender-based violence in workplaces is a major public health challenges and human rights violation worldwide. Yet there is a paucity of evidence on gender- based violence in higher educational institutions (HEIs) or so called Universities. The main aim of this study was to asses workplace gender-based violence and its associated factors among female staff in public HEIs in eastern Ethiopia.  Methods: Institutional-based cross-sectional study design was conducted among female staff working in public HEIs in Eastern Ethiopia. A total of 391 female staff were recruited using systematic sampling technique from three HEIs. Data were collected using adapted and pre-tested self-administered questionnaire that were facilitated by trained female data collectors. Collected data were entered into EpiData and exported to SPSS for cleaning and analysis. Descriptive statistics, binary and multivariable logistic regression analysis were carried out to compute frequencies and adjusted odds ratio (AOR) with 95% confidence interval (CI).  Results: Prevalence of workplace gender-based violence among female staff was 63.1% (95% CI: 58 – 68%). Being in the age group (18–34 years) (AOR:2.3, 95% CI:  1.11-4.91), being single (AOR:3.2, 95% CI: 1.07-9.74), divorced (AOR:3.7, 95% CI:  1.08-12.39), attained Bachelor Degree (AOR: 0.46, 95%: 0.24-0.90), work in night shift (AOR: 5.5, 95%CI: 1.65-15.77), not aware of the reporting procedures (AOR: 2, 95%CI: 1.23-3.33), and worried of violence (AOR: 1.71, 95%CI: 1.02-2.86), were the factors significantly associated with workplace gender-based violence against female staff in the public HEIs.  Conclusion: The prevalence of workplace gender-based violence among female staff working in the selected public HEIs was found to be unacceptably high. We suggest awareness- raising campaigns against gender-based violence, enforcing existing policies and procedures, orentation to employees, safe guarding night shifts workers, and support service for victims should be implemented by key stakeholders. |
| **Additional Information:** |  |
| **Question** | **Response** |
| **Financial Disclosure** | Haramaya University for financial support |

| Enter a financial disclosure statement that describes the sources of funding for the work included in this submission and the role the funder(s) played. This includes grants and any commercial funding of the work or authors.  This statement will be typeset if the manuscript is accepted for publication.  *Please review the* [*submission guidelines*](http://journals.plos.org/globalpublichealth/s/submission-guidelines#loc-financial-disclosure-statement) *and the instructions link below for detailed requirements and guidance.* |  |
| --- | --- |
| **Competing Interests**  On behalf of all authors, disclose any competing interests that could be perceived to bias this work.  This statement will be typeset if the manuscript is accepted for publication.  *Please review the instructions link below and PLOS Global Public Health’s* [*competing interests*](https://journals.plos.org/globalpublichealth/s/competing-interests) *policy to determine what information must be disclosed at submission.* | All authors declare that have no competing interest |
| **Data Availability**  Before publication, Authors are required to make fully available and without restriction all data underlying their findings. Please see our [PLOS Data](https://journals.plos.org/globalpublichealth/s/data-availability) [Policy](https://journals.plos.org/globalpublichealth/s/data-availability) page for detailed information on this policy.  A **Data Availability Statement**, detailing where the data can be accessed, is required at first submission. Insert your Data Availability Statement in the box below. | All related data are presented fully within the manuscript, and available up on reasonable request to the lead author (MAA: mowlid257@gmail.com) and the corresponding author (AS: agucell@yahoo.com) |

| Please see the [data reporting](https://journals.plos.org/globalpublichealth/s/submission-guidelines#loc-data-reporting) section of our submission guidelines for instructions on what you need to include in your Data Availability Statement.  This statement will be typeset if the manuscript is accepted for publication.  *PLOS allows rare exemptions to address legal and ethical concerns. If you have legal or ethical restrictions, please use the box below to detail these in full sentences for the Journal team to consider.* |  |
| --- | --- |

Manuscript	[Click here to access/download;Manuscript;Manuscript.docx](https://www.editorialmanager.com/pgph/download.aspx?id=191676&guid=c7f0b282-3d3f-42fc-9636-269b5051856d&scheme=1)

## 1 Workplace gender-based violence among female staff in public higher education

2 **institutions, eastern Ethiopia: Institution-based cross-sectional study**

3

4

5	Mowlid Abdi Ali^1^, Agumasie Semahegn ^2,3*^, Wondimye Ashenafi^1,4^, Henok Legesse ^2^

6

7

8

9 **Affiliation**
10 ^4^School of Public Health College of Health and Medical Sciences, Haramaya University,
11 Harar, Ethiopia
12 ^2^School of Nursing and Midwifery, College of Health and Medical Sciences, Haramaya
13 University, Harar, Ethiopia
14 ^3^Department of Population, Family and Reproductive Health, School of Public Health,
15 University of Ghana, Accra, Ghana
16 ^4^ National Data Management Center for Health (NDMC), Ethiopian Public Health Institute
17 (EPHI), Addis Ababa, Ethiopia

18

19 **Email**
20 MAA: [mowlid257@gmail.com](mailto:mowlid257@gmail.com)
21 AS: [agumas04@gmail.com](mailto:agumas04@gmail.com)
22 WA: [wondtg@gmail.com](mailto:wondtg@gmail.com)
23 HL: [henok_legesse@yahoo.com](mailto:henok_legesse@yahoo.com)

24

25

26

27 **Correspondence *:** Agumasie Semahegn ([agumas04@gmail.com](mailto:agucell@yahoo.com)); School of Nursing and
28 Midwifery, College of Health and Medical Sciences, Haramaya University, Harar, Ethiopia,
29 and Department of Population, Family and Reproductive Health, School of Public Health,
30 University of Ghana, Accra, Ghana

31

## 32 Abstract

33 **Introduction**: Gender-based violence in workplaces is a major public health challenges and
34 human rights violation worldwide. Yet there is a paucity of evidence on gender-based
35 violence in higher educational institutions (HEIs) or so called Universties. The main aim of
36 this study was to asses workplace gender-based violence and its associated factors among
37 female staff in public HEIs in eastern Ethiopia.

38

39 **Methods**: Institutional-based cross-sectional study design was conducted among female
40 staff working in public HEIs in Eastern Ethiopia. A total of 391 female staff were recruited
41 using systematic sampling technique from three HEIs. Data were collected using adapted
42 and pre-tested self-administered questionnaire that were facilitated by trained female data
43 collectors. Collected data were entered into EpiData and exported to SPSS for cleaning and
44 analysis. Descriptive statistics, binary and multivariable logistic regression analysis were
45 carried out to compute frequencies and adjusted odds ratio (AOR) with 95% confidence
46 interval (CI).

47

48	**Results**: Prevalence of workplace gender-based violence among female staff was 63.1% 49	(95% CI: 58 – 68%). Being in the age group (18–34 years) (AOR:2.3, 95% CI: 1.11-4.91), 50 being single (AOR:3.2, 95% CI: 1.07-9.74), divorced (AOR:3.7, 95% CI: 1.08-12.39),

51 attained Bachelor Degree (AOR: 0.46, 95%: 0.24-0.90), work in night shift (AOR: 5.5,
52 95%CI: 1.65-15.77), not aware of the reporting procedures (AOR: 2, 95%CI: 1.23-3.33),
53 and worried of violence (AOR: 1.71, 95%CI: 1.02-2.86), were the factors significantly
54 associated with workplace gender-based violence against female staff in the public HEIs.

55

56 **Conclusion:** The prevalence of workplace gender-based violence among female staff
57 working in the selected public HEIs was found to be unacceptably high. We suggest
58 awareness- raising campaigns against gender-based violence, enforcing existing policies and
59 procedures, orentation to employees, safe guarding night shifts workers, and support service
60 for victims should be implemnted by key stakeholders.

61

62 **Keywords**: Gender-based violence, female staff, Higher Educational Institutions, Ethiopia

## 63 Introduction

64 According to the 1993 United Nations (UN), gender-based violence is defined as “any act
65 of violence that results in, or is likely to result in, physical, sexual, or mental harm or
66 suffering to women, including threats of such acts, coercion or arbitrary deprivation of
67 liberty, whether occurring in public or in private life” (1). Violence against women at
68 workplace perhaps is the most pervasive, but less recognized human rights abuse in the world
69 (1, 2). Workplace gender-based violence (GBV), in its different forms (physical, sexual and
70 psychological) is worldwide problem that affect large number of women at their work sites
71 (2, 3)(4, 5). Majority of women reported for being not safe and victim of different forms of
72 workplace GBV (5, 6).

73

74 Workplace GBV is recognized as a severe human rights abuse and major public health
75 challenge with substantial consequences on physical, mental, and reproductive well beings
76 of female staff (7-12). Globally, 38% of women were murdered by their intimates (1), and
77 the extent of workplace GBV ranging from 10% to 69% worldwide (4), and huge inequities
78 found across regions. The prevalence of GBV was 20% in the Western Pacific, 22% in
79 Europe, 33% in the WHO African region (1), and 67.7% in sub-Saharan Africa that include
80 Ethiopia (9). Workplace GBV in higher education setting is alarmingly rising and almost
81 becoming a growing concern (13). As a result of the unsafe and male dominancy in the HEIs,
82 female staff are at increased risk to workplace GBV (14). However, the causes of workplace
83 GBV are multi-dimensional where the interplay of social, economic, cultural, political and
84 religious factors affects the victimization and perpetration. Besides, senior male co-workers
85 are the main perpetrators at workplaces due to power imbalance (15-17).

86

87 Workplace GBV is a significant public health issue and a human rights agenda in Ethiopia.
88 A systematic review and meta-analysis in Ethiopia revealed that the workplace violence is
89 high and it is exceptionally high among female university (HEIs) staff (18). However, there
90 is a paucity of evidence on factors influencing workplace GBV which could be the main
91 reason for the poor implementation of workplace violence prevention and control programs
92 in Ethiopia (18). Therefore, we conducted an institution-based study that aimed at assessing
93 workplace GBV and its associated factors among female staff in public HEIs in eastern
94 Ethiopia.

95

# 96 Materials and methods

## 97 Study setting

98 The study was conducted in three public universities or HEIs in the eastern part of Ethiopia,
99 namely: Haramaya University, Dire Dawa University and Jigjiga University, from
100 December 1 to 30 December, 2022. Haramaya University is located in Haramaya town,
101 eastern Ethiopia that is the second oldest Public HEIs in Ethiopia. It was established in 1954
102 as ‘Alemaya College of Agriculture’ under Addis Ababa University until 1985. Currently,
103 Haramaya University has widely been engaged in the expansion and diversification of
104 undergraduate, graduate and postgraduate trainings (19). Dire Dawa University (DDU) is a
105 public university located in Dire Dawa, Ethiopia that is founded in 2007.as second-
106 generation university, and has six colleges (20). Similarly, Jigjiga University (JJU) is one of
107 the public University that was established in 2007 second-generation universities in
108 Ethiopia. It’s found in Jigjiga, the capital of the Somali Region, Ethiopia. Jigjiga is located,
109 635 kilometers from Addis Ababa, eastern Ethiopia. It has 8 faculties; it also offers both post
110 and under graduate program (21). Most of the populations are pastoralists as well as engaged
111 in trade, cultivation of cash crops, and in transitive life (22).

## 113 Study design and participants

114 An institutional-based cross-sectional study was conducted on the selected public
115 universities. All female staff whose age above 18 years and who had been working for at
116 least 6 months in the selected universities were included in the study. Nevertheless, female
117 staff who were on annual leave, maternity leave and severe health conditions during data
118 collection period were excluded from the study.

## 120 Sampling procedures

121 Sample size was determined by using single population proportion formula considering
122 parameters of workplace violence prevalence (63.8%) in Nigeria (17), 95% significance
123 level, 1% margin of error, and adding 10% potential non-response compensation. Finally,
124 the sample size was 391. Then the calculated sample size was proportionally allocated to the
125 number of female staff at selected public HEIs (**Fig 1**). The sampling frame was constructed
126 by listing all female staff working in the selected public HEIs. Actual study participants were
127 selected using systematic sampling. The first study participant from each university was
128 selected by using lotters method and then every participant (k^th^) was selected according to
129 the respective sampling interval until the sample size was fulfilled for each university.
130 **Fig 1:** Schematic presentation of sampling procedures

## 132 Data collection procedure

133 Data were collected using self-administered structured questionnaire that was adapted from
134 International Labor Office (2), country based survey questionnaire on workplace violence
135 and from “UNICEF: Ending gender based violence survey questionnaire” on HEIs (23). The
136 questionnaire contains: socio-demographic and workplace characteristics (12 items),
137 Institutional/contextual characteristics (9 items), GBV assessing questions for psychological
138 violence (8 items), physical violence (4 items), and sexual violence and/or harassment (14
139 items) and responses toward the incidents (5 items). It was first written in English and then
140 translated to Amharic, Afaan-Oromo and Somali language and was re-translated to English
141 by language experts to ensure consistency. Data collection were facilitated by 10 trained
142 female Nurses. Four data collectors for Haramaya University, three for Jigjiga University,
143 three for Dire Dawa University were assigned to facilitate the self-administered
144 questionnaire distribution, clarification as deemed necessary and re-collection. Training was
145 provided to data collectors on the study objectives, sampling, consent, data privacy, checking
146 for data clarity and completeness by the principal investigator. Then data collectors taken
147 informed consent from the study participants.

## 149 Outcome variable measurements

150 Workplace GBV against female staff at HEIs was measured as a composite variable using
151 the three very common types of workplaces GBV, namely physical violence, psychological
152 violence and sexual violence and/or harassment. So, in this study, workplace GBV was
153 considered if a female staff (15-49 years) reported experienced of at least one forms of
154 violence (physical, psychological, or sexual violence) by male co-worker, supervisor/boss,
155 visitor or strange person at their workplace in the past 12 months (10). Psychological
156 violence includes either one of: intentional use of power, including threat of physical force,
157 act of verbal abuse including insults and bullying or mobbing. Physical violence was
158 assessed when female staff answered “yes” to one of the items: slapping, kicking, beating
159 with any object, cutting, pushing, shoving, throwing, strangling, grabbing or pulling, and
160 shooting against female staff. Sexual violence is defined as acts of violence at workplace
161 that are done on a female staff by intentional use of physical force or power, intimidation or
162 threatening (making to fear) to have sex or to engage in acts of sex without the consent. It
163 includes completed rape, attempted rape and sexual harassment.

## 165 Data processing and analysis

166 Collected data were entered into Epi-Data 3.1 and exported to IBM SPSS version 25 for
167 cleaning and analysis. Descriptive statistics was carried to determine the proportion, mean
168 with standard deviations of explanatory variables and prevalence of workplace violence.
169 Binary and multiple logistic regression analyses were performed to examine the association
170 between the independent variables and workplace GBV. Explanatory variables with a p-
171 value < 0.25 in binary logistic regression analysis were included in the multivariable logistic
172 regression analysis. The Hosmer-Lemeshow test was used for goodness of fit for the final
173 model fitness. The adjusted odds ratio (AOR) with 95% confidence interval (CI) at P-Value
174 of < 0.05 was considered to declare significance association between explanatory variables
175 and outcome variable.

# 177 Results

## 178 Socio-demographic characteristic of study participant

179 A total of 385 study participants completed the questionnaire (Fig.1) which made the
180 response rate 98.5% (385/391). The mean age for participants was 32.39 (SD±8.65) years.
181 Nearly half of them were married (45.2%, n=174) and belonging to Orthodox Christianity
182 (48.3%, n=186). Forty percent (n=151) of the study participants were bachelor degree
183 holders (Table 1).
185 Table 1. Socio-demographic characteristics of the study participants in HEIs, eastern

186	Ethiopia, 2022 (n=385)

| **Variables** | **Categories** | n | **%** |
| --- | --- | --- | --- |
| Age | 18-34 | 246 | 63.9 |
|  | ≥35 | 139 | 36.1 |
| Marital status | Single | 146 | 37.9 |
|  | Married | 174 | 45.2 |
|  | Divorced | 43 | 11.2 |
|  | Widowed | 22 | 5.2 |
| Religion | Muslim | 107 | 27.8 |
|  | Orthodox | 186 | 48.3 |
|  | Protestant | 78 | 20.3 |
|  | Others* | 14 | 3.6 |
| Ethnicity | Oromo | 138 | 35.8 |

|  | Amhara | 160 | 41.6 |
| --- | --- | --- | --- |
|  | Somali | 61 | 15.8 |
|  | Others** | 26 | 6.8 |
| Educational status | Secondary school | 35 | 9.1 |
|  | Diploma level | 113 | 29.4 |
|  | Bachelor’s degree | 154 | 40 |
|  | Master’s degree and above | 83 | 21.5 |
| Salary level (Ethiopian Birr) | 1100-2344 | 117 | 30.4 |
|  | 2799-5358 | 117 | 46.0 |
|  | 6193-10150 | 71 | 18.4 |
|  | ≥10150 | 20 | 5.2 |
| Service years | 1-4 | 163 | 42.3 |
|  | 5-9 | 146 | 37.3 |
|  | ≥10 | 76 | 19.7 |
| Residence | Inside campus | 70 | 18.2 |
|  | Outside campus | 315 | 81.8 |

187 ∗*Others include: Catholic, Waqefata, Jehovah’s Witness, un-affiliated.* ∗∗*Others include: Tigray, Gurage,*
188 *Sidama, Harari*

## 189 Workplace related characteristics

190 Out of 385, nearly half (47.5%, n=183) of them were from Haramaya University. Majority
191 (90.4%) of them did not work in night shifts, and 71.7% of them did not know how to report
192 violence. Slightly more than three-fourth (76.3%, n=293) of them were administrative staff,
193 and nearly all of them (96.4%) had permanent employment (Table 2).
195 Table 2: Institutional related characteristics of female staff working in public HEIs of
196 eastern Ethiopia, 2022 (n=385)

| **Variables** | **Categories** | n | **%** |
| --- | --- | --- | --- |
| Institution | HU | 183 | 47.5 |
|  | JJU | 98 | 25.5 |
|  | DDU | 104 | 27 |
| Staff category | Academic | 92 | 23.9 |
|  | Non- Academic | 293 | 76.3 |
| Number of co- workers | 1-4 | 307 | 79.7 |
|  | 5-9 | 45 | 11.7 |
|  | ≥10 | 33 | *8 .6* |
| Night shift | Yes | 37 | 9.6 |
|  | No | 348 | 90.4 |
| Employment status | Temporary | 14 | 3.6 |
|  | Permanent | 371 | 96.4 |
| Reporting  procedures | Yes | 109 | 28.3 |
|  | No | 276 | 71.7 |
| Institutional policy | Yes | 235 | 61 |
|  | No | 150 | 39 |
| Worried of violence | Not worried | 134 | 34.8 |
|  | Little worried | 50 | 13 |

|  | Moderately worried | 143 | 37.1 |
| --- | --- | --- | --- |
|  | Much worried | 58 | 15.1 |

## 198 Workplace gender-based violence against female staff

199 Female staff experience of workplace GBV was computed as a composite measure. By
200 considering any forms of psychological, sexual and physical violences, the prevalence of
201 workplace GBV against female staff in HEIs was 63.1% (95% CI: 58-68%) (Fig 2).
202 Fig **2**: Workplace GBV among female staff in public HEIs, eastern Ethiopia, 2022 (n=385)

## 204 Perpetrators of the workplace GBV

205 One-third (34.8%) of female staff perpetrated by their supervisor/boss, followed by 20.8%
206 of them perpetrated by co-workers (Fig. 3).
207 Fig **3**: Perpetrators of workplace GBV among female staff in public universities of eastern

208	Ethiopia, 2022 (n=385)

209

210 More than a quarter (28.1%) of female staff who had experienced workplace GBV reported
211 that reduced productivity due to GBV at workplace. More than one-in-ten (11.7%) of them
212 stay off work after the incident. One-in-five (20.5%) of them claimed that they were
213 emotionally exhausted after experienced violence at their workplace.

## 215 Factors associated with workplace gender-based against female staff

216 In the binary logistic regressions, a total of 20 independent variables were included. Then
217 only 11 variables namely; marital status, educational status, residence, work experiences,
218 number of co-workers, age, staff category, night shift, reporting procedure, worried of
219 violence and institutional policy were found to be significant with a p-value of < 0.25 in the
220 bivariate analysis (Table 3). In the final multivariable logistic regression model, six variables
221 remained significantly associated with workplace GBV. Being single (AOR: 3.2, 95% CI:
222 1.07-9.74) and separated/divorced (AOR: 3.7, 95% CI: 1.08-12.39) were associated with
223 increased odds of WPGBV as compared to being currently married. Moreover, being in age
224 group of 18-34 years (AOR: 2.3, 95% CI: 1.11-4.91), working in the night shift (AOR:5.5,
225 95% CI: 1.65-15.77), being not aware of the existence of reporting procedures (AOR: 2.0,
226 95% CI: 1.23-3.33) and being worried about violence (AOR: 1.71, 95% CI: 1.02-2.86) were
227 also associated with increased odds of WPGBV as compared with their counterparts. On the
228 other hand, the odds of experiencing workplace GBV were decreased by 54% among female
229 staff with BSc degrees compared to female staff with Masters and above degree (AOR:0.46,

230	95% CI:0.24-0.90) (Table 3).

231

232 Table 3. Factors associated with GBV among female staff working in public HEIs in the
233 eastern Ethiopia, 2022 (n=385)

| **Variable** | **Category** | **Workplace GBV** | | **COR (95%CI)** | **AOR (95% CI)** |
| --- | --- | --- | --- | --- | --- |
|  |  | **Yes** | **No** |  |  |
| Marital status | Single | 110 | 36 | 4.4(1.74-11.2) | 3.2(1.07 -9.74) * |
|  | Widowed | 9 | 13 | 2.1(0.67-4.12) | 1.4(0.49-4.17) |
|  | Divorced | 31 | 12 | 3.7(1.31-10.1) | 3.7(1.08-12.39) * |
|  | Married | 93 | 81 | 1 | 1 |
| Educational Status | ≤Secondary | 23 | 12 | 0.61(0.26-1.44) | 0.74(0.28- 1.97) |
|  | Diploma | 76 | 37 | 0.65(0.34-1.24) | 0.86(0.42-1.78) |
|  | BSc | 81 | 73 | 0.35(0.19-0.64) | 0.46(0.24-0.90) * |
|  | ≥MSc | 63 | 20 | 1 | 1 |
| Residence in campus | Inside | 49 | 21 | 1.45(0.83-2.55) | 1.5 (0.79-2.87) |
|  | Outside | 194 | 121 | 1 | 1 |
| Work experience | 1-4 | 95 | 68 | 1.02(0.59-1.76) | 0.73(0.39-1.37) |
|  | 5-9 | 104 | 42 | 1.8(1.01-3.22) | 1.32(0.70-2.52) |
|  | ≥10 | 44 | 32 | 1 | 1 |
| Co-workers’ | 1-4 | 183 | 124 | 0.4(0.17-0.94) | 0.61(0.23-1.59) |
|  | 5-9 | 34 | 11 | 0.8(0.28-2.44) | 1.20(0.38-3.99) |
|  | ≥10 | 26 | 7 | 1 | 1 |
| Age | 18-34 | 171 | 75 | 2.7 (1.4-5.1) | 2.30(1.11-4.91) * |
|  | ≥35 | 50 22 | 66 | 1 | 1 |
| Staff category | Academic  Administrative | 63  180 | 29  113 | 1.4 (0.8-2.3)  1 | 1.30(0.74-2.43)  1 |
| Night shift | Yes | 33 | 4 | 0.2 (0.1-0.5) | 5.5(1.65-15.77) * |
|  | No | 210 | 138 | 1 | 1 |
| Reporting procedure | Yes | 188 | 88 | 1 | 1 |
|  | No | 55 | 54 | 2.1 (1.33-3.29) | 2(1.23-3.33) ***** |
| Worried of violence | Worried | 94 | 40 | 0.69(0.1-0.44) | 1.71(1.02-2.86) * |
|  | Not worried | 149 | 102 | 1 | 1 |
| Institutional  policy | Yes | 101 | 49 | 1 | 1 |
|  | No | 142 | 93 | 0.7(0.48-1.14) | 0.79 (0.49-1.29) |

234 **Significantly associated at a p-value of <0.05*

## 236 Discussion

237 This study determined the prevalence of workplace GBV and associated factors among
238 female staff in public higher education institutions. Nearly two-third (63.1%, 95%CI: 58–
239 68%) of female staff at HEIs experienced workplace GBVs during the last 12 months. This
240 finding is in line with the study conducted in Nigeria, where prevalence of workplace GBV
241 among female staff was (63.8%) (24), and the study conducted in Iran (63.2%) (25).
242 However, it is higher than the findings in Nepal (49.5%) (6), in Turkey (44.7%) (8), in
243 Rwanda (58.5%) (10), in Ethiopia (35.1%) (26). This discrepancy is might be due to nature
244 of the study, cultural, socio-economic difference and it might also be due to violence
245 prevention regulations, strategies and policies existed in those countries. The findings of this
246 study is lower than the finding of study conducted in China (77.5%) (27), and a comparative
247 study on gender based violence in educational institutions of Kenya (70%) (28).
249 In the present study psychological violence is the most common forms of workplace GBV
250 (57.7%). This findings is similar with the study conducted in Nigeria (29), Gambia (30).
251 Different forms of psychological violence were faced by the female staff once and more than
252 twice times in last twelve months in higher education institutions. In the present study sexual
253 violence found to be (14.5%), this finding is similar with study conducted in Nepal (6).
254 Sexual violence of this findings is much lower than the study conducted in Ethiopia that
255 have reported 49.1% in Bahir Dar City 49.1% (31), 39.2% in Dessie City (32), 56.4% in
256 Harar (33). The finding of this study showed that sexual violence high in younger age female
257 staff compared to older age, the study is similar with study conducted in Ethiopia (34).
258 Similarly, the findings of the present study revealed that (28.3%) of female staff were
259 experienced physically violence which is similar with study conducted in Zimbabwe (35),
260 study conducted in Harar town, Ethiopia, where nearly one-third of women experienced at
261 least one form of physical violence in Ethiopia (33)(36) and in United Kingdom (14).
263 According to the findings of the current survey having bachelor degree significantly
264 associated with less experience of workplace GBV. This result is in line with study
265 conducted Taiwan (37) and in Nigeria among female staff university also support this finding
266 (24). Different findings might indicate that education protects women against disrespectful
267 or inappropriate behavior at work, including in academics and the findings (38, 39). The
268 findings of this study revealed that female staff between (18-34) years experienced more
269 GBV at workplace. This result is similar with the study conducted in Ethiopia (3).
270 Furthermore, our findings showed that as age increased the risks of GBV at workplaces
271 among university female staff decreases. This findings may be that people’s reverence for
272 old age in various Ethiopia cultures discourages the exhibition of violence against older
273 female staff, this finding is supported with study conducted in Nigeria (24), as well as study
274 conducted in China (40).
276 In the present study, participants work Night shifts was 5.5 times were more likely to
277 experiences workplace GBV. This findings was similar to the study conducted in Egypt (41)
278 and the study conducted in Ethiopia among female staff at Mekelle University (42), public
279 hospitals of Eastern Ethiopia (43). The odds of workplaces GBV were higher in those
280 females who were not-aware of reporting procedure compared to those female staff who
281 aware reporting procedures. These factors were noted in a study conducted in Saudi Arabia
282 (44) and study conducted in Ethiopia (36). The finding of this study demonstrated that being
283 worried about violence at workplace were substantially associated with Workplaces GBV.
284 These factors were in line with the study conducted in Brazil (45). This might be indicating
285 that those who were worried of violence were working in a predisposed work environment.

## 287 Strength and weakness of the study

288 The study used a validated and comprehensive tool that encompasses the magnitude, nature
289 and effect of workplace GBV in a relatively broader fashion. But there is the possibility of
290 response biases since research evidence suggests that female staff tend not to report
291 workplace of GBV experience for fear of stigma or retribution. It may also be prone to recall-
292 bias. The causal relationship between explanatory variables and workplace GBV may be
293 limited due to temporality challenges in the cross-sectional study design.

## 295 Conclusions

296 The prevalence of workplace GBV among female staff at higher education institution, in
297 eastern Ethiopia was found to be high. Female staff experience of forms of GBV in the
298 workplace was significantly associated with their age, lower academic qualification, marital
299 status, reporting procedures and work experience of 4-9 years. It was mainly perpetrated by
300 supervisors. Therefore, it’s recommended to establish workplace GBV committee that would
301 track the incidents, provide guidelines and make sure all corrective measures were taken and
302 communicated properly.

## 304 Acronyms and abbreviations

305 AOR: Adjusted Odds Ratio; CI: Confidence Interval; COR: Crude Odds Ratio; GBV:
306 Gender-Based Violence; HEI: Higher Education Institutions; KII: Key Informant Interview;
307 UN: United Nations; VAW: Violence Against Women; WHO: World Health Organization;
308 WPV: Workplace Violence; WPGBV: Workplace Gender-Based Violence

## 309 Declaration

310 **Ethical consideration**
311 The study protocol was reviewed and approved by Institutional Health Research Ethical
312 Review Committee College of Health and Medical Science, Haramaya University, (Refn.:
313 IHRERC/176/2022). A formal letter was submitted for each university administrations and
314 permission was granted to conduct the data collection. The study was conducted in
315 accordance with the declaration of Helsinki’s. Informed verbal and written consent were
316 obtained from each study participant in voluntary basis to be included in the study. Informed
317 consent from participant/legal guardian and assent from them were obtained from study
318 participants whose age younger than 18 years old, and also not attended formal education.
319 Collected data had kept confidential anonymously through de-identification of names and
320 other personal identifiers from record/sheet., parents/guardians in case of minor study
321 participants and legally authorized representatives in case of illiterate participants.

## 322 Competing interests: Authors have no competing interest

323 **Consent for publication:** Not applicable
324 **Availability of data and materials:** All related data are presented fully within the
325 manuscript, and available up on reasonable request to the lead author (MAA:
326 [mowlid257@gmail.com](mailto:mowlid257@gmail.com)) and the corresponding author (AS: [agucell@yahoo.com](mailto:agucell@yahoo.com)).
327 **Competing interests:** All authors declare that have no conflict of interest.
328 **Funding:** Haramaya University provided the financial and administrative supports for this
329 study.

## 330 Authors’ Contributions

331 MAA, AS, and WA had involved since conception, study design, execution, and acquisition
332 of data, analysis and interpretation. AS, HL and MAA drafted the manuscript. All authors
333 revised the manuscript critically for important intellectual contents. All authors read and
334 approved the final manuscript.

## 335 Acknowledgments

336 We would like to acknowledge Haramaya University for financial support, and the study
337 participants, data collectors, supervisors, health facility administrators, and staff for their
338 willingness to give their time and information for this study.

## 339 Authors information

340 (MAA & WA)^1^ School of Public Health College of Health and Medical Sciences, Haramaya
341 University, Harar, Ethiopia (AS &, HL)^2^School of Nursing and Midwifery, College, Health
342 and Medical Sciences, Haramaya University, Harar, Ethiopia; (AS)^3^Department of
343 Population, Family and Reproductive Health, School of Public Health, University of Ghana,
344 Accra, Ghana;

## 345 References

346 1.	WHO. Violence against women prevalence estimates, 2018: global, regional and national prevalence
347 estimates for intimate partner violence against women and global and regional prevalence estimates for non-

348	partner sexual violence against women. ISBN 978-92-4-002225-6 2021;ISBN 978-92-4-002225-6 112.

349	2.	ILO W, WHO PJGI. Framework guidelines for addressing workplace violence in the health sector.

350	2002.

351 3.	Tiruneh BT, Bifftu BB, Tumebo AA, Kelkay MM, Anlay DZ, Dachew BAJBn. Prevalence of
352 workplace violence in Northwest Ethiopia: a multivariate analysis. 2016;15(1):1-6.
353 4.	Garcia-Moreno C, Amin AJS, matters rh. Violence against women: where are we 25 years after ICPD
354 and where do we need to go? 2019;27(1):346-8.
355 5.	Adebayo O, Ugorji J, Ekeh A, Jolade E, Odoh N, Ikeme A, et al. The impact of workplace violence
356 on nurses and nursing practice: A literature review. 2022.
357 6.	Heise L EM, Gottemoeller M. Ending Violence against Women. Baltimore: John’s Hopkins
358 University School of Public Health; Population Information Program; 1999. Report No.: Series L, No. 11.
359 Gender based violence at workplace: are women safe. 2014.
360 7.	Fute M, Mengesha ZB, Wakgari N, Tessema GAJBn. High prevalence of workplace violence among
361 nurses working at public health facilities in Southern Ethiopia. 2015;14(1):1-5.
362 8.	Pinar T, Acikel C, Pinar G, Karabulut E, Saygun M, Bariskin E, et al. Workplace Violence in the
363 Health Sector in Turkey: A National Study. Journal of interpersonal violence. 2017;32(15):2345-65.
364 9.	Mingude AB, Dejene TM. Prevalence and associated factors of gender based violence among Baso
365 high school female students, 2020. Reproductive health. 2021;18(1):247.
366 10.	Musengamana V, Adejumo O, Banamwana G, Mukagendaneza MJ, Twahirwa TS, Munyaneza E, et
367 al. Workplace violence experience among nurses at a selected university teaching hospital in Rwanda. 2022;41.
368 11.	Sabbar DK, Kassim WJJPJoM, Sciences H. Relationship between Workplace Related Violence and
369 Job Satisfaction among Nurses Staff. 2022;16(03):1018-.
370 12.	Teshome E, Kumar S. GENDER-BASED VIOLENCE AND ITS ASSOCIATION WITH MENTAL
371 HEALTH AMONG WOMEN WITH DISABILITIES. ISSN (Online): 0493-2137. 2022;Vol:55 Issue.
372 13.	Mahlori XF, Byrne DC, Mabude LAJSo. Perceptions of gender-based violence among university
373 staff: interim results. 2018;8(3):21.
374 14.	Jones C, Skinner T, Gangoli G, Smith O, Fenton RJTSCWP. Gender-based Violence Among UK
375 University Students and Staff: A Socio-Ecological Framework. 2020;21(21-2).
376 15.	Esther O. GENDER-BASED VIOLENCE IN WORKPLACES: A CRITICAL EXAMINATION OF
377 ITS IMPLICATIONS IN THE CONTEMPORARY SOCIETY. 2020;1(2).
378 16.	Oppong D, Bannor RKJCSS. Gender and power work relationships;: A systematic review on the
379 evidence from Africa and Asia. 2022;8(1):2031686.
380 17.	Olaoluwa Samson Agbaje1 CKA, Joshua Emeka Umeifekwem1,, Prince Christian Iheanachor
381 Umoke1* CCI, Tochi Emmanuel Iwuagwu1, Cylia Nkechi Iweama1, Eyuche Lawretta Ozoemena1 and Edith
382 N. Obande‑ Ogbuinya2. Workplace gender-based violence and associated factors among university
383 women in Enugu, South-East Nigeria: an institutional-based cross-sectional study. 2021;21(1):1-20.
384 18.	Worke MD, Koricha ZB, Debelew GTJRh. Prevalence of sexual violence in Ethiopian workplaces:
385 systematic review and meta-analysis. 2020;17(1):1-15.
386 19.	Mesele TT, Dheresa M, Oljira L, Wakwoya EB, Gemeda GMJIJoWsH. Prevalence of dysmenorrhea
387 and associated factors among Haramaya university students, eastern Ethiopia. 2022:517-27.
388 20.	Haileyesus M, Meretu D, Abebaw EJJoE, Development S. Determinants of university staff turnover
389 intention: The case of Dire Dawa university, Ethiopia. 2019;10(17):20-9.
390 21.	Crump L, Mohammed Ali S, Tschopp R, Zinsstag JJOHCS. Jigjiga University One Health Initiative.

391	2022(2022):ohcs20220001.

392 22.	Mureda E, Zeleke ZMJH. Characteristics and constraints of crossbred dairy cattle production in
393 lowland areas of Eastern Ethiopia. 2008;21(47.6):33.3.
394 23.	Ranea-Triviño B, Pajares L, Bustelo M, Pereira BCJ, PU DL. Report on Case Studies on the Effects
395 and Consequences of Institutional Responses to Gender-based Violence along the 7Ps in Research Performing
396 Organisations.
397 24.	Olaoluwa Samson Agbaje1 CKA, Joshua Emeka Umeifekwem1,, Prince Christian Iheanachor
398 Umoke1* CCI, Tochi Emmanuel Iwuagwu1,, Cylia Nkechi Iweama1 ELOaENOO. Workplace gender-based
399 violence and associated factors among university women in Enugu, South-East Nigeria: an institutional-based
400 cross-sectional study. 2021;21(1):1-20.
401 25.	Nasrabadi AN, Abbasi NH, Mehrdad NJGjohs. The prevalence of violence against Iranian women
402 and its related factors. 2015;7(3):37.
403 26.	Birkie M, Zenebe Y, Biset G, Gebresellassie M, Mihret SJTOPHJ. Risk factors for gender-based
404 violence among female students of Gonder Teacher’Training College, Gonder, Northwest Ethiopia: a cross-
405 sectional study. 2020;13(1).
406 27.	Sui G, Liu G, Jia L, Wang L, Yang G. Associations of workplace violence and psychological capital
407 with depressive symptoms and burn-out among doctors in Liaoning, China: a cross-sectional study. BMJ open.

408	2019;9(5):e024186.

409 28.	Venâncio KC, da Fonseca RM. [Women working at university restaurants: life and work conditions
410 and gender-based violence]. Revista da Escola de Enfermagem da U S P. 2013;47(5):1016-24.
411 29.	Iliyasu Z, Abubakar IS, Aliyu MH, Galadanci HS, Salihu HMJAjorh. Prevalence and correlates of
412 gender-based violence among female university students in Northern Nigeria. 2011;15(3):123-33.
413 30.	Sisawo EJ, Ouédraogo SYYA, Huang S-LJBhsr. Workplace violence against nurses in the Gambia:
414 mixed methods design. 2017;17(1):1-11.
415 31.	Belay HG, Liyeh TM, Tassew HA, Ayalew AB, Goshu YA, Mihretie GNJIjorm. Magnitude of
416 gender-based violence and its associated factors among female night students in Bahir Dar City, Amhara

417	Region, Ethiopia. 2021;2021:1-7.

418	32.	Gebrie S, Wasihun Y, Abegaz Z, Kebede NJBwsh. Gender-based violence and associated factors

419	among private college female students in Dessie City, Ethiopia: mixed method study. 2022;22(1):513.

420	33.	Abubeker F, Dessie Y, Assefa N, Geleto A, Adorjan K, Abdeta TJITJoHCO, Provision,, et al.

421	Prevalence and associated factors of gender-based violence among female students attending private colleges

422	in Harar Town, Eastern Ethiopia. 2021;58:00469580211047197.

423 34.	Henock A, Deyessa N, Abajobir AJJWH, Issues Care 4. Sexual violence and substance use among
424 female students of Mizan-Tepi University, Southwest Ethiopia: A mixed method study. 2015;4(2).
425 35.	Mukanangana F, Moyo S, Zvoushe A, Rusinga OJAjorh. Gender based violence and its effects on
426 women’s reproductive Health: the case of Hatcliffe, Harare, Zimbabwe. 2014;18(1):110-22.
427 36.	Legesse H. Workplace Violence And Its Associated Factors Among Nurses Working In Government
428 Hospitals Of Harari Regional State And Dire Dawa City Administration, Eastern Ethiopia: Haramaya
429 university; 2021.
430 37.	Yoo M, Lee S, Kang MY. Gender and educational level modify the relationship between workplace
431 mistreatment and health problems: a comparison between South Korea and EU countries. Journal of

432	occupational health. 2015;57(5):427-37.

433	38.	Albezreh S, Anastario M, Ulibarrí BJ, Naimer K, Johnson K, McHale T, et al. Multiyear, Multisectoral

434	Training Program in Kenya to Enhance Medical–Legal Processes in Response to Sexual and Gender-Based

435	Violence. 2022;28(14):3311-30.

436	39.	Bondestam F, Lundqvist MJEJoHE. Sexual harassment in higher education–a systematic review.

437	2020;10(4):397-419.

438	40.	Jan AJIJoTiSR, Development. Violence against Women with Special Reference to Domestic Violence

439	Act, 2005. 2019;3(3):1398-401.

440	41.	Abou-ElWafa HS, El-Gilany A-H, Abd-El-Raouf SE, Abd-Elmouty SM, El-Sayed Hassan El-Sayed

441	RJJoiv. Workplace violence against emergency versus non-emergency nurses in Mansoura university

442	hospitals, Egypt. 2015;30(5):857-72.

443	42.	Galu SB, Gebru HB, Abebe YT, Gebrekidan KG, Aregay AF, Hailu KG, et al. Factors associated with

444	sexual violence among female administrative staff of Mekelle University, North Ethiopia. 2020;13(1):1-7.

445	43.	Legesse H, Assefa N, Tesfaye D, Birhanu S, Tesi S, Wondimneh F, et al. Workplace violence and its

446	associated factors among nurses working in public hospitals of eastern Ethiopia: a cross-sectional study.

447	2022;21(1):1-10.

448	44.	Alkorashy HAE, Al Moalad FBJInr. Workplace violence against nursing staff in a Saudi university

449	hospital. 2016;63(2):226-32.

450	45.	Tsukamoto SAS, Galdino MJQ, Robazzi MLdCC, Ribeiro RP, Soares MH, Haddad MdCFL, et al.

451	Occupational violence in the nursing team: prevalence and associated factors. 2019;32:425-32.

452

Figure	[Click here to access/download;Figure;Fig 1.docx](https://www.editorialmanager.com/pgph/download.aspx?id=187524&guid=db23f960-325e-429a-9b76-3a3d367aae1c&scheme=1)

Proportional allocation

Eligible

**Fig 1:** Schematic presentation of sampling procedures

*DDU: Dire Dawa University, HrU: Haramaya University, and JJU: Jigjiga University*

n= 391

105

101

185

DDU

1852

JJU 1765

HrU

3234

Total female staff at 3 HEIs (N=6,851)

included

Figure	[Click here to access/download;Figure;Fig 2.docx](https://www.editorialmanager.com/pgph/download.aspx?id=187525&guid=1d98291f-f57a-4d68-8e80-b059d9190075&scheme=1)

70

60

50

40

30

20

10

0

63.1%

57.7%

28.3%

14.5%

Prevalence of

workplace gender- based violence

Psychological violence

physical violence

sexual violence

Fig **2**: Workplace gender-based violence among female staff working in public HEIs universities of eastern Ethiopia, 2022 (n=385)

Figure	[Click here to access/download;Figure;Fig 3.docx](https://www.editorialmanager.com/pgph/download.aspx?id=187526&guid=2d8ae585-9304-4b7c-bddc-24553a8218e4&scheme=1)

40

35

30

25

20

15

10

5

0

34.8%

20.8%

5.8%

1.7%

Boss/supervisor colleagues/other

staff

Student

Strangers

Perpetrators

Fig **3**: Perpetrators of workplace GBV among female staff in public universities of eastern Ethiopia, 2022 (n=385)
