# Supplementary material for: Workplace gender-based violence among female staff in public higher education institutions in eastern Ethiopia: Institution-based cross-sectional study
Source: PLOS Glob Public Health. 2024 Jul 26;4(7):e0003489. doi: 10.1371/journal.pgph.0003489 (PMC11280138; doi:10.1371/journal.pgph.0003489)
Supplement: Attachment — Submitted filename: point by point response Plos Global Public Health.docx [file pgph.0003489.s005.docx]

**Response to Reviewers comments**

To: **PLOS Global Public Health**

Title: *Workplace gender-based violence among female staff in public higher education institutions, eastern Ethiopia: Institution-based cross-sectional study
PLOS Global Public Health [PGPH-D-23-01225]*
Dear Editor and reviewers;

We have received comments from reviewers and we fund them constructive and helpful. We have addressed the reviewers’ concern point-by-point here below

General comment

Thank you for submitting your manuscript to PLOS Global Public Health. After careful consideration, we feel that it has merit but does not fully meet PLOS Global Public Health’s publication criteria as it currently stands. Therefore, we invite you to submit a revised version of the manuscript that addresses the points raised during the review process.
**Comments to the Author**
1. Does this manuscript meet PLOS Global Public Health’s [publication criteria](https://journals.plos.org/globalpublichealth/s/criteria-for-publication)? Is the manuscript technically sound, and do the data support the conclusions? The manuscript must describe methodologically and ethically rigorous research with conclusions that are appropriately drawn based on the data presented.

Reviewer #1: Yes

Reviewer #2: Partly: *We have made revision, hope it is to the standard now*.

2. Has the statistical analysis been performed appropriately and rigorously?

Reviewer #1: Yes

Reviewer #2: Yes

3. Have the authors made all data underlying the findings in their manuscript fully available (please refer to the Data Availability Statement at the start of the manuscript PDF file)?

The [PLOS Data policy](https://journals.plos.org/globalpublichealth/s/data-availability) requires authors to make all data underlying the findings described in their manuscript fully available without restriction, with rare exception. The data should be provided as part of the manuscript or its supporting information, or deposited to a public repository. For example, in addition to summary statistics, the data points behind means, medians and variance measures should be available. If there are restrictions on publicly sharing data—e.g. participant privacy or use of data from a third party—those must be specified.

Reviewer #1: Yes

Reviewer #2: Yes

4. Is the manuscript presented in an intelligible fashion and written in standard English?

PLOS Global Public Health does not copyedit accepted manuscripts, so the language in submitted articles must be clear, correct, and unambiguous. Any typographical or grammatical errors should be corrected at revision, so please note any specific errors here.

Reviewer #1: Yes

Reviewer #2: Yes

*-Response: Many thanks. We have revised our manuscript as per the recommendation.*

| Section | Reviewers’’ comment | ***Authors’ response*** |
| --- | --- | --- |
| Journal Requirements | 1. We noticed you have some minor occurrence of overlapping text with the following previous publication(s), which needs to be addressed:  - <https://www.yada.org.tr/> - <https://doi.org/10.1186/s12905-022-02001-8> | ***Many thanks for the comment. we made some re-phrasing to avoid overlapping of terms and phrases for previous publications.*** |
|  | **Reviewer #1:** |  |
|  | The manuscript is very well written and touched a very important & sensitive issue. For more and clear information I, have attached the file. The manuscript need minor revision. How the participants were contacted and how questionnaire were distributed and collected back should be discussed in detail. | ***We are so grateful for the complement, and address the concerns raised as well. See the revised manuscript with track change for visibility of the changes*** |
|  | The green and yellow highlighted comments | ***Many thanks for the constructive comment and we have gone page by page and addressed one-by-one. See track change*** |
|  | **Reviewer #2:** |  |
|  | On the grounds that much of the literature on GBV is from a western perspective, this paper should be considered for publication. Yet it needs major revision, in my opinion. | ***Many thanks for the comment, we have addressed the comments forwarded from the attached documents as follow;*** |
| Abstract | Abstract The study resulted that women were not aware how to report in case of violence | ***thanks, Corrected as per suggestion. See track change: pg- 2*** |
| Introduction | - Stick to the workplace | ***many thanks, corrected as per suggestion*** |
| Method | - Methods: Participants and eligibility criteria | ***we have addressed. We consider employees work at least 6 months is due to university employment modality. To be a staff, employees should finish the contract period for the first 6 months and participants before that period may not give adequate information in confidence due to being on provisional period. Otherwise, they may even more vulnerable during that period.*** |
|  | - Sample size formula | ***The sample size calculation formula is added n = (𝑍𝛼/2)^2^*𝑃(1−𝑃) / 𝑑^2^ and Female staff whose age above 18 were eligible for the study, and sample of 391 female staff were included in the study*** |
|  | Please elaborate on how the questionnaires were distributed and collected back. How data privacy was assured: | ***The self-administered questionnaire was distributed in envelope and re-collected the filled-out questionnaire through*** |
| Other general comments | 1. The language needs attention – I have highlighted in green the spelling errors, sentence construction errors, incorrect word usage or tenses that I spotted as I reviewed. Proof reading will be required on the final submission | ***Many thanks for the constructive comment and we have addressed all the highlighted in yellow and green, typos, and grammer page by page and addressed one-by-one. See track change*** |
|  | 2. The reporting of other studies, and indeed the authors own findings need to be reported in more detail and with the degree of precision required for an academic journal. Simple comparisons of percentages are only valid if the authors can clarify that the same measurement instruments and cut off points were employed across studies. Otherwise the comparisons are of limited utility. This needs to addressed | ***Many thanks for the comment. we have made massive revision. Hope your concerns are now addressed substantially and fit to the journal requirement and /or standard. See track change*** |
|  | 3. There is no contextual information on the cultural backdrop to the study. Why are these rates so high? What are other HEIs doing to address this problem? Can the Ethiopian Universities use similar approaches? The data as presented is of interest but the discussion lacks depth and consideration. A more probing and insightful analysis and discussion is required for publication. There is virtually no reference to the large international literature on this topic | ***Many thanks for the comment.***  ***We have added reference related to contextual evidence in responding and preventing gender-based violence. See track change*** |
|  | 4. There is an implicit assumption that GBV is violence perpetrated by men, upon women. This is a limited view and persons who do not conform to a stereotypical sex role or gender identity may be a victim of GBV. Authors need to demonstrate knowledge of the nuances and complexities of this area. | ***Many thanks for the comment. The study was actual explored in the way. See revised version for the modifications.*** |

*Many thanks!*

*Corresponding Author*
