# Supplementary material for: Workplace gender-based violence among female staff in public higher education institutions in eastern Ethiopia: Institution-based cross-sectional study
Source: PLOS Glob Public Health. 2024 Jul 26;4(7):e0003489. doi: 10.1371/journal.pgph.0003489 (PMC11280138; doi:10.1371/journal.pgph.0003489)
Supplement: Attachment — Submitted filename: point by point response Plos Global Public Health.docx [file pgph.0003489.s006.docx]

**Response to Reviewers comments**

To: **PLOS Global Public Health**

Title: *Workplace gender-based violence among female staff in public higher education institutions, eastern Ethiopia: Institution-based cross-sectional study
PLOS Global Public Health [PGPH-D-23-01225]*
Dear Editor and reviewers;

We have received comments from reviewers and we fund them constructive and helpful. We have addressed the reviewers’ concern point-by-point here below

| Section | Reviewers’’ comment | ***Authors’ response*** |
| --- | --- | --- |
|  | **Reviewer #1:** | ***Many thanks for the critical review and relevant recommendations for improvement*** |
|  | Author has done all the changes as suggested in last revision. Manuscript has touched a very important and sensitive area | ***Many thanks for the constructive comments so far.*** |
|  | Reviewer #3: |  |
|  | Thank you for inviting me to review this interesting research area: gender I have the following concerns and feedback on the work: 1. Definition and approach: though I agree that females are disproportionately affected by violences, I dont agree that concept gender is not all about females. There should be points that put more males at risk than females sometimes. Studying females alone is possible but not the only way. I would encourage the authors to clearly define gender violence and appreciate other rrlavant terms such as- gender vs sex vs sex orientation, sexual violence, pyschological violence, physical violences too. How do you see studying gender(social definition) but sampling female participants ( biological definition). In the UN definition you considered the word "women". Who are women in your study? Biologically females? Does it include those who consider themselves as women though biologically male at birth? Are there anyone who consider themselves as women while being male at birth in the study setting? How did you know? Do you have preminary assessment about this? Or have you checked how "females" included in this study consider themselves? What if they consider themselves as men? Overall, do not consider definiton of gender superficially because it leads to superficial responses or findings. Please improve your background by clearly out those points by writing in the manuscript. | ***Many thanks for the comments. Sure, gender stands for both male and female but female are more disproportionately victimized due to social status and social constructions. We considered women who have been employed by the Universities as female staff. Such kind of sexual orientation is not well recognized and very sensitive in the context of Ethiopia. Thank you for the comment.*** |
| 2. Methods | measurement is one of the methodological issues not well addressed in this study. I found that this study has almost no new finding. It is okay to repeat studies but efforts should be there to add new knowledge to literatute. Measurement issues of this study include: | ***Thanks, this study is among the very few evidences available in Ethiopia, even in sub-Saharan countries. It will have great contribution to literature and knowledge.*** |
|  | 1) how this study understands gender and how it measured key aspects of gender is a big limitation. Gender has sociocultural, organizational, systematic or structural factors or drivers. Did you assess empowerment (education, economy, leadership roles), decision making, etc.? That is not reflected. Both qualitativr and wuantitativr methods was required to shade light on those things-how they shaped "gender violences". | ***Thank you so muchf or the comment. This study is mainly intended to measure the violence, but did not address the gender disparity /gap issues. It is the limitation.*** |
|  | 2) the meaurement of sexual violence is superficial. How did you measure and report sexual harassment? Please report such things as reported ones because harasment is too subjective to define. That may be the reason why the estimate of violence is as high as 63% in your study. Sexual violence has 3 aspects in this study: rape, attempted rape, and harasment. Better to report these separately for the readers to know the estimates for each. | ***Many thanks for the comment. Specific items results are now added to he manuscript to give a little bit detailed about the measurement used for three major type of GBV.*** |
|  | 3) pyschological violence is more blurred in this study. Please define, measure, and report them clearly. Please provide proper descriptions for each items included to measure it. This should also be done for sexual and physical aspects too. Now it is over merged. Please give % of each items in the description section. For associated factor analysis you may used merged or latent variable. | ***Many thanks for the comment. Specific items results are now added to he manuscript to give a little bit detailed about the measurement used for three major type of GBV.*** |
|  | 4) why qualitative data are missed study given its great relevance? | ***Thanks for the suggestion. GBV is relatively not new study. So quantitative study might is required*** |
|  | 3: Discussion and conclusion: properly discuss gender violence interms of sociocultural and systemic factors. I see a great deal of limitations in this accord that should be clearly and honestly indicated in this manuscript. I hope reanalysis of gender violence and its specific aspects will provide different estimate. | ***Many thanks for the comment, and well noted and addressed the comments. Hope you are happy with the revised version*** |
|  | 4: general question: have you reported this huge estimate of violence to your institution leaderships and collected their remarks? It can also be considered as qualitative data and even reported in this study. | ***Many thanks for the suggestion and we will consider the suggestion of qualitative study in the future*** |

*Many thanks!*

*Corresponding Author*
